# Targeting apoptosis; design, synthesis and biological evaluation of new benzoxazole and thiazole based derivatives

**DOI:** 10.1186/s13065-023-01101-2

**Published:** 2024-01-03

**Authors:** Sama W. Helmy, Mai I. Shahin, Nermin Samir, Deena S. Lasheen, Dalal A. Abou El Ella

**Affiliations:** Pharmaceutical Chemistry Department, Faculty of Pharmacy, Ain Shams University, African Union Organization St. Abbassia, Cairo, 11566 Egypt

**Keywords:** Apoptosis, Bcl-2, Caspase, Benzoxazole, Thiazole, Colorectal cancer

## Abstract

Several novel approaches to target Bcl-2 proteins and apoptotic pathways have been identified in recent years for the treatment of different types of cancer including colorectal cancer. However, no effective treatments were yet developed for colorectal cancer. Twenty two novel benzoxazole and thiazole−based compounds were designed, synthesized, and evaluated as potential Bcl-2 inhibitors with anti−proliferative activity. Compounds **8g, 12e** and **13d** showed good to moderate anti−proliferative activity against most of the NCI 60 cell line panel with mean growth inhibition percent of 45.13, 42.29 and 29.25%, respectively. They showed the greatest cell growth inhibition percent to HCT-116 cell line with the values of 68.0, 59.11 and 43.44%, respectively. The aforementioned compounds were furtherly investigated for their effect on HCT-116 cell cycle, and they showed increase in the total apoptosis with 17, 22, and 5%, respectively. Also, the apoptotic effect of compounds **8g, 12e** and **13d,** were tested by their effect on altering caspase-3 expression level in HCT-116 human cell line. The three compounds showed an increase in the caspase-3 levels by 6, 8 and 3 folds, respectively in comparison with the same untreated ones. Moreover, they were evaluated for their* in–vitro* Bcl-2 inhibitory activity and they showed percent inhibition of 60.2, 69.2 and 50.0%, respectively. Finally, the most potent compounds **8g** and **12e** showed 3.864 and 2.834 folds increase in Bax level compared to the control respectively. On the other hand, Bcl-2 was down−regulated to 0.31 and 0.415 folds compared to the control. The induction of apoptosis through increase in caspase 3 expression and down−regulation of Bcl-2 is the suggested mechanism of action.

**Supplementary Information:**

The online version contains supplementary material available at 10.1186/s13065-023-01101-2.

## Introduction

Apoptosis is a Greek term used to describe the situation in which a cell actively pursues a course toward death upon receiving certain stimuli [1]. Being a highly selective process, apoptosis is important in both physiological and pathological conditions. The main specific feature of apoptosis is the activation of a group of enzymes belonging to the cysteine protease family named caspases. Activated caspases cleave many vital cellular proteins and break up the nuclear scaffold and cytoskeleton. They also activate DNAase, which further degrade nuclear DNA [2]. There are three pathways by which caspases can be activated. The two commonly known initiation pathways are the intrinsic (or mitochondrial) and extrinsic (or death receptor) pathways of apoptosis. Both pathways eventually lead to a common pathway or the execution phase of apoptosis [3].

The Bcl-2 family of proteins is comprised of pro−apoptotic and anti−apoptotic proteins. They play a pivotal role in the regulation of apoptosis, especially via the intrinsic pathway as they reside upstream of irreversible cellular damage and act mainly at the mitochondrial level. Both the anti−apoptotic and pro−apoptotic functions of Bcl-2 family members are regulated through their BH domains. Furthermore, the BH1-BH3 domains of anti−apoptotic proteins form a hydrophobic binding pocket that binds the α-helix of the BH3-only pro−apoptotic protein [4]. These protein–protein interactions govern cell fate. In cancer, there is a disruption in the balance between anti−apoptotic and pro−apoptotic members of the Bcl-2 family. This can be due to an overexpression of one or more anti−apoptotic proteins or an under−expression of one or more pro−apoptotic proteins or a combination of both. This imbalance leads to sequestering the pro−apoptotic proteins by the anti−apoptotic ones resulting in a dysregulated apoptosis.

Evasion of cell death is one of the essential changes that cause a normal cell to be transformed into a malignant one or cancerous cell [5]. Hence, reduced apoptosis or its resistance plays a vital role in carcinogenesis and that was observed in many types of cancers including colorectal cancer. Though colorectal cancer is one of the leading causes of cancer deaths worldwide, no effective treatments were yet developed to efficiently treat it [6]. 5-FU is the drug used for patients with colorectal cancer. It is expected by 2030 to have 2.2 million new cases and 1.1 million deaths recorded annually [7]. Bcl2 was found to be highly expressed in colorectal cancer tissues which subsequently leads to lower apoptosis rates. This reveals the importance of targeting Bcl-2 and other apoptosis related targets to treat such fatal disease [8].

In this context, twenty two analogues based on benzoxazole and thiazole heterocycles were designed, synthesized, and evaluated for their anticancer activities against NCI 60-cell line panel. The design was based on comprehensive SAR study of previously reported Bcl-2 inhibitors. The good activity shown against Bcl-2 and HCT-116 cell line along with the compliance of the scaffolds synthesized pave the way towards further optimization to have a library of analogues with promising anti−proliferative activities.

### Rationale and design

**Fig. 1:**
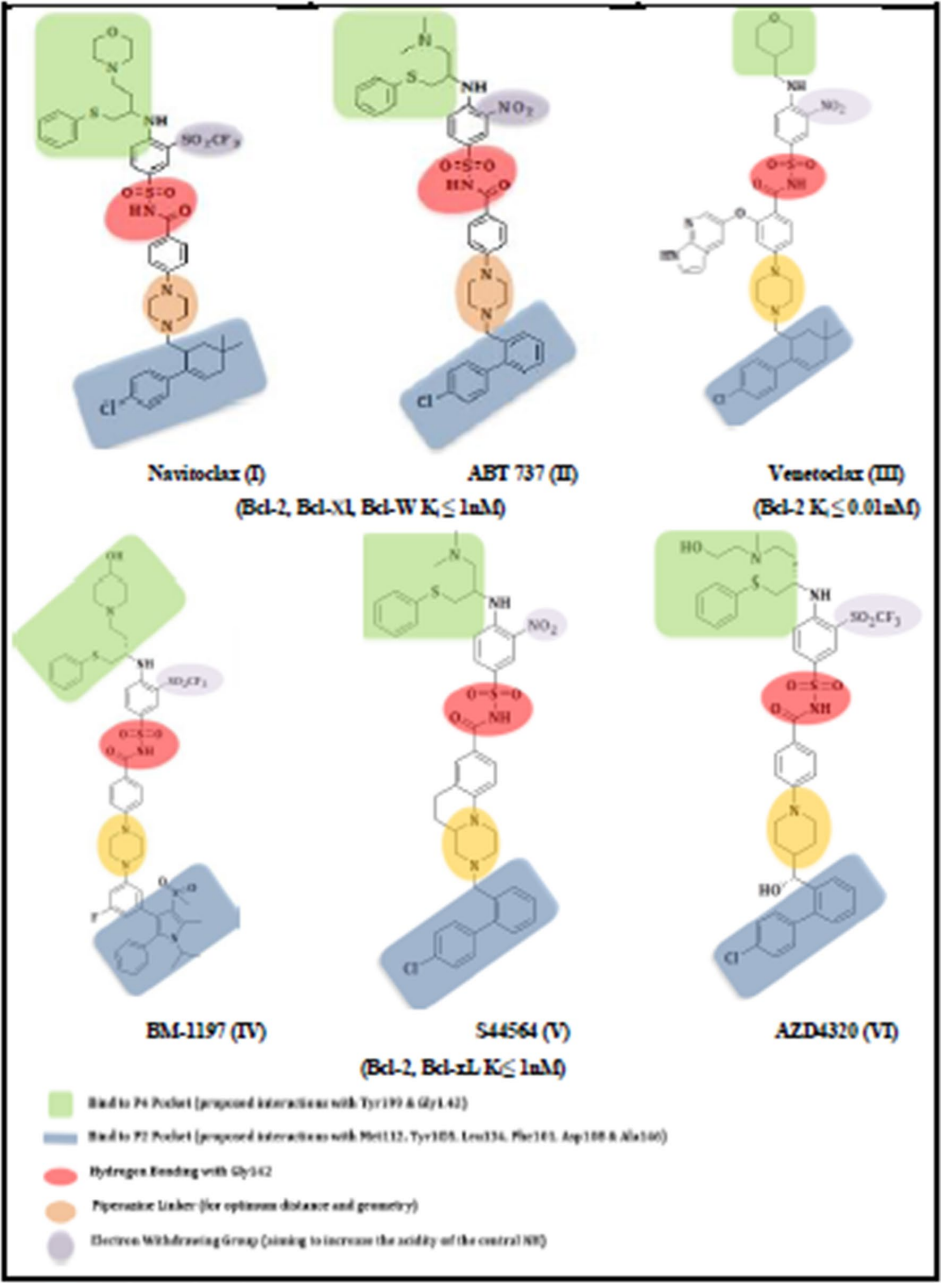
Common pharmacophoric features of some Bcl-2 inhibitors and proposed interactions with Bcl-2 binding site

The current study aims to design and synthesize novel series of benzoxazole and thiazole−based compounds targeting Bcl-2 anti−apoptotic proteins. The design depends on the reported common features and SAR studies of known Bcl2 inhibitors (Fig. 1).

**Fig. 2:**
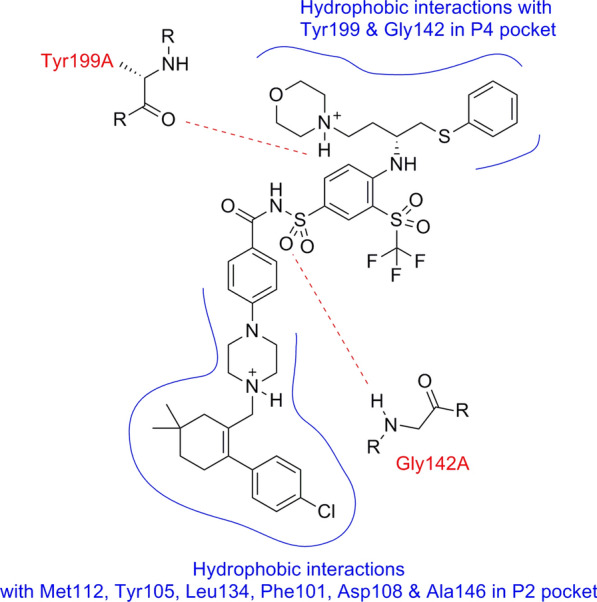
Key interactions of Navitoclax (I) with Bcl-2

The co−crystal structure of Navitoclax **(I)** generally reveals three main pharmacophoric features. The first feature is chlorophenyl cyclohexene component which is located in the P2 pocket and contributes to the activity on both Bcl-2 and Bcl−xL. The second feature consists of phenyl thioether moiety which is linked to the tri−substituted phenyl and binds deeply within the P4 pocket. The third one involves the central 4-piperazinyl-N-arylsulfonylbenzamide component which provides the key H−bonding with Gly142 amino acid residue and also represents essential pharmacophoric feature for Bcl-2 inhibitory activity [9] (Fig. 2).

The synthesized compounds were obtained from the optimization of lead compound **(I)** depending on the following strategies: (1) Central to the structure of Navitoclax **(I)** is an acyl sulfonamide moiety, which acts as linker between the two pockets binding moieties and forms the key hydrogen bonding with Gly142. Therefore, a structural modification approach was applied to test the effect of bioisosteric amide, expecting to capture the same hydrogen bonding with Bcl-2 binding site. (2) The acidic nature of the NH of acyl sulfonamide has been shown to impact the potency, solubility, and clearance rates of these compounds [10], so in an attempt to increase the amide acidity, a different structural modification approach was proposed by changing the nearby phenyl fragment into heterocyclic rings introducing new benzoxazole and thiazole scaffolds. (3) Efforts to downsize the P4 interaction moiety into smaller primary or secondary amine derivatives were also conducted to decrease the molecular weights of the designed compounds making them more synthetically feasible and increasing their drug−likeness. (4) The piperazine moiety was conserved to other heterocyclic linkers in the designed compounds.

**Fig. 3:**
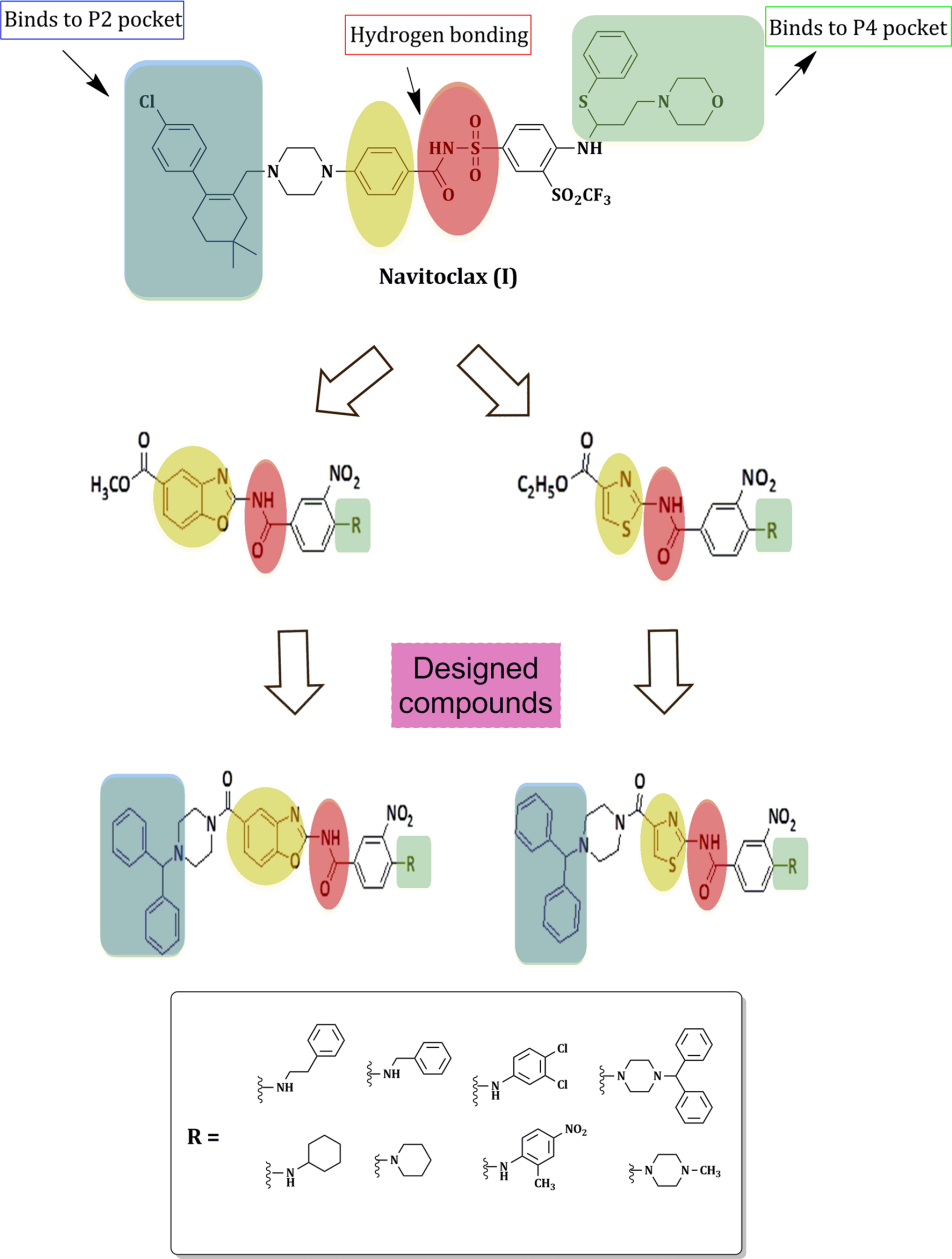
Design of novel Bcl-2 inhibitors

As it is a protein–protein interaction, blocking either the P2 or P4 pocket is expected to be enough for inhibiting the anti−apoptotic activity of Bcl-2 [11] (Fig. 3).

## Materials and methods

### Chemistry and analysis

Starting materials, reagents and solvents were purchased from Sigma−Aldrich (USA), Alfa−Aesar, Loba Chemie Organics, Fisher scientific or Sigma−Aldrich and were used without further purification. Column chromatography was conducted on silica 60 (35–70 microns). Melting points were recorded on a Stuart SMP10 melting point apparatus. IR spectra (KBr) were recorded on a Perkin−Elmer spectrophotometer.1H-NMR spectra were recorded on a Bruker 400 MHz spectrometer in δ scale (ppm), using DMSO as solvent at special unit facility, Faculty of Pharmacy, Ain−shams University. EI−MS spectra were recorded by Triple Quadrupole LC/Ms/Ms mass spectrometer API 200 (AB Sciex Instrument) at the regional center of mycology and biotechnology et al.−Azhar University, Egypt. Elemental analyses were determined at the same center utilizing FLASH 2000 CHNS/O analyzer, Thermo Scientific. Compounds **(1, 2, 3, 4, 5, 6a−c, 6e, 6g, 6h, 7a−c, 7e, 7g and 7h)** were prepared according to the reported procedures [12–20].

### 4-Substituted-3-nitrobenzoic acid (6a-h)

#### General procedure

To a suspension of compound **5** (2 g, 10 mmol) in 20 mL DMF, anhydrous K_2_CO_3_ (1.65 g, 12 mmol) was added. Different primary and secondary amines (10 mmol) were added portion wise while cooling then the reaction mixture was allowed to stir overnight at room temperature. The resulting solutions were poured on ice−cold water giving different precipitates which were filtered and washed with small quantity of cold water to give the titled compounds **(6a**-**h)**, yield 55–65%.

### 4-(2-Methyl-4-nitrophenylamino)-3-nitrobenzoic acid (6d)

The titled compound was obtained as yellow solid; 60% yield; m.p. 182–184 °C;**1H NMR (400 MHz, DMSO**-***d6)*** δ 11.02 (s, 1H, OH), 7.78 (s, 1H, ArH), 7.74 (d, J = 7.3 Hz, 1H, ArH), 7.69 (d, J = 7.5 Hz, 1H, ArH), 7.66 (d, J = 7.5 Hz, 1H, ArH), 7.62 (d, J = 7.2 Hz, 1H, ArH), 7.58 (s, 1H, ArH), 2.36 (s, 3H, CH3).

### 4-(4-Benzhydrylpiperazin-1-yl)-3-nitrobenzoic acid (*6f*)

The titled compound was obtained as white solid; 63% yield; m.p. 238–240 °C;**1H NMR (400 MHz, DMSO-d6)** δ 11.05 (s, 1H, OH), 7.74 (s, 1H, ArH), 7.68 (d, J = 7.3 Hz, 1H, ArH), 7.59 (d, J = 7.5 Hz, 1H), 7.52–7.41 (m, 10H, phenyl), 4.95 (s, 1H, CH), 2.55 (s, 4H, piperazine), 2.32 (s, 4H, piperazine).

### 4-Substitued-3-nitrobenzoyl chloride (7a−h)

#### General procedure

The respective acid derivative **(6a-h)** (1 g) was dissolved in 15 mL dry DCM then thionyl chloride (5 mL) was added dropwise while cooling. The reaction mixture was refluxed for 7 h. The completion of the reaction was confirmed using TLC. Then, the remaining solvent was evaporated in vacuo to give compounds **(7a-h)**, yield 50–60%. These compounds were used in the next step directly without further purification.

### Methyl 2-[N-(4-substituted-3-nitrobenzamido)]benzo[*d*].oxazole-5-carboxylate (8a-g)

#### General procedure

To a solution of compound **3** (0.5 g, 2.6 mmol) in 10 mL dry DCM and 3 drops of TEA, the respective benzoyl chloride derivative (**7a-g) (**2.6 mmol) in 10 mL dry DCM was added dropwise while cooling. The reaction mixtures were stirred at room temperature for 24–48 h. The completion of the reactions was confirmed using TLC. The resulting solutions were poured on ice−cold water (30 mL) giving different precipitates which were filtered and washed with small quantity of cold water to give the titled compounds **(8a-g)**, yield 20–60%. They were purified by column chromatography using hexane: ethyl acetate gradient elution (9:1–1:1) and confirmed by 1H NMR spectroscopy.

### Methyl 2-[N-(3-nitro-4-(phenethylamino)benzamido)]benzo[*d*]oxazole-5-carboxylate (8a)

The titled compound was separated as buff crystals (31%); m.p. 222–224 °C;^**1**^**H NMR (400 MHz, DMSO-d6)** δ 10.57 (s, 1H, NH), 8.58 (s, 1H, ArH), 7.94 (dd, *J* = 7.5 Hz, 2H, ArH), 7.77–7.50 (m, 5H, phenyl), 7.45 (s, 1H, ArH), 7.29 (dd, *J* = 7.8 Hz 2H, ArH), 3.84 (s, 3H, OCH3), 3.21–2.65 (m, 4H, CH2CH2). **MS** (Mwt = 460.44) m/z (% rel. Int.), 460.14 (M+, 100%). **Anal.** Cacld for C24H20N4O6: C, 62.60; H, 4.38; N, 12.17; Found: C, 62.82; H, 4.56; N, 12.00.

### Methyl 2-[N-(4-benzylamino-3-nitrobenzamido)]benzo[*d*]oxazole-5-carboxylate (8b)

The titled compound was separated as offwhite crystals (39%); m.p. 173–175 °C; ^**1**^**H NMR (400 MHz, DMSO-d6)** δ 10.35 (s, 1H, NH), 7.95 (s, 1H, ArH), 7.93 (s, 1H, ArH), 7.77 (d, *J* = 7.7 Hz, 1H, ArH), 7.75 (d, *J* = 7.5 Hz, 1H, ArH), 7.58 (d, *J* = 7.3 Hz, 1H, ArH), 7.56 (d, *J* = 7.4 Hz, 1H, ArH), 7.21 (s, 5H, phenyl), 3.86 (s, 3H, OCH3), 3.05 (s, 2H, CH2).** MS** (Mwt= 446.41) m/z (% rel. Int.), 446.12 (M+, 100%). **Anal.** Cacld for C23H18N4O6: C, 61.88; H, 4.06; N, 12.55; Found: C, 60.84; H, 3.65; N, 12.23.

### Methyl-2-[N-(4-(3,4-dichloroanilino)-3-nitrobenzamido)]benzo[*d*]oxazole-5-carboxylate (8c)

The titled compound was separated as buff crystals (22%); m.p. 150–153°C; ^**1**^**H NMR (400 MHz, DMSO-d6)** δ 10.62 (s, 1H, NH), 8.16 (s, 1H, ArH), 7.79 (s, 1H, ArH), 7.75 (d, *J* = 7.6 Hz, 1H, ArH), 7.63 (d, *J* = 7.7 Hz, 1H, ArH), 7.61 (d, *J* = 7.2 Hz, 1H, ArH), 7.57 (d, *J* = 8.3 Hz, 1H, ArH), 7.41 (d, *J* = 8.6 Hz, 1H, ArH), 7.12 (s, 1H, ArH), 6.88 (d, *J* = 8.3 Hz, 1H, ArH), 3.86 (s, 3H, OCH3). **MS** (Mwt= 501.28) m/z (% rel. Int.), 502.03 (M^+^+2, 33.31%), 501.03 (M++1, 30.79%), 500.03 (M+, 100%). **Anal.** Cacld for C22H14Cl2N4O6: C, 52.71; H, 2.82; N, 11.18; Found: C, 52.55; H, 2.45; N, 11.36.

### Methyl-2-[N-(4-(2-methyl-4-nitroanilino)-3-nitrobenzamido)]benzo[*d*]oxazole-5-carboxylate (8d)

The titled compound was separated as yellow crystals (22%); m.p. 158–160 °C; ^**1**^**H NMR (400 MHz, DMSO-d6)** δ 10.34 (s, 1H, NH), 8.04 (s, 1H, ArH), 7.86 (d, *J* = 7.5 Hz, 1H, ArH), 7.83 (d, *J* = 7.7 Hz, 1H, ArH), 7.81 (d, *J* = 7.6 Hz, 1H, ArH), 7.78 (d, *J* = 7.6 Hz, 1H, ArH), 7.76 (d, *J* = 7.7 Hz, 1H, ArH), 7.62 (d, *J* = 8.6 Hz, 1H, ArH), 7.60 (s, 1H, ArH), 7.58 (s, 1H, ArH), 3.86 (s, 3H, OCH3), 2.11 (s, 3H, CH3).** MS** (Mwt= 491.41) m/z (% rel. Int.), 491.11 (M^+^, 100%). **Anal.** Cacld for C23H17N5O8: C, 56.22; H, 3.49; N, 14.25; Found: C, 55.84; H, 3.65; N, 13.23.

### Methyl 2-[N-(4-cyclohexylamino-3-nitrobenzamido)]benzo[*d*]oxazole-5-carboxylate (8e)

The titled compound was separated as buff crystals (21%); m.p. 160–163 °C; ^**1**^**H NMR (400 MHz, DMSO-d6)** δ 10.47 (s, 1H, NH), 7.93 (d, *J* = 8.1 Hz, 1H, ArH), 7.80 (s, 1H, ArH), 7.77 (d, *J* = 8.5 Hz, 1H, ArH), 7.58–7.54 (m, 3H, ArH), 3.85 (s, 3H, OCH3), 1.24–1.15 (m, 11H, cyclohexane).** MS** (Mwt= 491.41) m/z (% rel. Int.), 491.11 (M+, 70%), 367.26 (100%). **Anal.** Cacld for C22H22N4O6: C, 60.27; H, 5.06; N, 12.78; Found: C, 60.50; H, 5.26; N, 13.05.

### Methyl-2-[N-(4-(4-benzhydrylpiperazin-1-yl)-3-nitrobenzamido)]benzo[*d*]oxazole-5-carboxylate (8f)

The titled compound was separated as buff crystals (60%); m.p. 174–176 °C; ^**1**^**H NMR (400 MHz, DMSO-d6)** δ 10.35 (s, 1H, NH), 7.96 (s, 1H, ArH), 7.74 (s, 1H, ArH), 7.66 (dd, *J* = 7.3 Hz, 2H, ArH), 7.49–7.27 (m, 10H, phenyl), 7.19 (dd, *J* = 7.5 Hz, 2H), 4.75 (s, 1H, CH), 3.84 (s, 3H, OCH3), 2.70 (s, 4H, piperazine), 2.22 (s, 4H, piperazine).** MS** (Mwt = 591.61) m/z (% rel. Int.), 591.21 (M+, 80%), 167.24 (100%). **Anal.** Cacld for C33H29N5O6: C, 67.00; H, 4.94; N, 11.84; Found: C, 67.23; H, 5.25; N, 11.66.

### Methyl-2-[N-(4-(4-methylpiperazin-1-yl)-3-nitrobenzamido)]benzo[*d*]oxazole-5-carboxylate (8 g)

The titled compound was separated as white crystals (54%); m.p. 151–153 °C; ^**1**^**H NMR (400 MHz, DMSO-d6)** δ 11.25 (s, 1H, NH), 8.80 (s, 1H, ArH), 7.96 (d, *J* = 7.1 Hz, 1H, ArH), 7.44 (d, *J* = 7.2 Hz, 1H, ArH), 7.32 (d, *J* = 7.7 Hz, 1H, ArH), 7.29 (s, 1H, ArH), 7.21 (d, *J* = 7.4 Hz, 1H, ArH), 4.36 (s, 3H, OCH3), 2.41–2.24 (m, 8H, piperazine), 1.24 (s, 3H, CH3).** MS** (Mwt = 439.42) m/z (% rel. Int.), 439.01 (M+, 65%), 367.74 (100%). **Anal.** Cacld for C21H21N5O6: C, 57.40; H, 4.82; N, 15.94; Found: C, 57.22; H, 5.02; N, 15.77.

### Ethyl 2-[N-(4-substituted-3-nitrobenzamido)]thiazole-4-carboxylate (9a−h)

#### General procedure

To a solution of compound **4** (0.5 g, 2.9 mmol) in 10 mL dry DCM and 3 drops of TEA, the appropriate benzoyl chloride derivative **(7a-h)** (2.9 mmol) in 10 mL dry DCM was added dropwise while cooling. The reaction mixtures were stirred at room temperature for 24–48 h. The completion of the reactions was confirmed using TLC. The resulting solutions were poured on ice−cold water giving different precipitates which were filtered and washed with small quantity of cold water to give the titled compounds **(9a-h)**, yield 20–60%. They were purified by column chromatography using hexane: ethyl acetate gradient elution (9:1–1:1) and confirmed by 1H NMR spectroscopy.

### Ethyl 2-[N-(3-nitro-4-(phenethylamino)benzamido)]thiazole-4-carboxylate (9a)

The titled compound was separated as white crystals (33%); m.p. 170–172 °C; ^**1**^**H NMR (400 MHz, DMSO-d6)** δ 13.10 (s, 1H, NH), 8.66 (s, 1H, thiazole), 8.12 (s, 3H, phenyl), 7.8 (s, 1H, ArH), 7.61 (d, 2H, phenyl), 7.5 (d, *J* = 7.8 Hz, 1H, ArH), 7.18 (d, *J* = 7.5 Hz, 1H, ArH), 4.30 (q, 2H, OCH2), 2.85–2.51 (m, 4H, CH2CH2), 1.31 (t, 3H, CH3). **MS** (Mwt= 440.47) m/z (% rel. Int.), 440.12 (M+, 100%). **Anal.** Cacld for C21H20N4O5S: C, 57.26; H, 4.58; N, 12.72; Found: C, 57.44; H, 4.38; N, 12.77.

### Ethyl 2-[N-(4-benzylamino-3-nitrobenzamido)]thiazole-4-carboxylate (9b)

The titled compound was separated as buff crystals (42%); m.p. 110–112 °C; ^**1**^**H NMR (400 MHz, DMSO-d6)** δ 10.47 (s, 1H, NH), 8.22 (s, 1H, thiazole),7.94 (d, *J* = 8.4 Hz, 1H, ArH), 7.63 (s, 1H, ArH), 7.57 (d, *J* = 8.5 Hz, 1H, ArH), 7.25 (s, 5H, phenyl), 4.27 (q, 2H, OCH2), 3.06 (s, 2H, CH2), 3.04 (t, 3H, CH3). **MS** (Mwt= 426.45) m/z (% rel. Int.), 426.10 (M+, 50%), 172.22 (100%). **Anal.** Cacld for C20H18N4O5S: C, 56.33; H, 4.25; N, 13.14; Found: C, 56.65; H, 4.44; N, 13.02.

### Ethyl 2-[N-(4-(3,4-dichloroanilino)-3-nitrobenzamido)]thiazole-4-carboxylate (9c)

The titled compound was separated as brown crystals (35%); m.p. 186–188 °C; ^**1**^**H NMR (400 MHz, DMSO−d6)** δ 10.25 (s, 1H, NH), 8.02 (s, 1H, thiazole), 7.60 (d, *J* = 8.6 Hz, 1H, ArH), 7.55 (s, 1H, ArH), 7.53 (s, 1H, ArH), 7.34 (d, *J* = 7.5 Hz, 1H, ArH), 7.09 (d, *J* = 7.4 Hz, 1H, ArH), 7.07 (d, *J* = 7.5 Hz, 1H, ArH), 4.28 (q, 2H, OCH2), 3.05 (t, 3H, CH3). **MS** (Mwt= 481.31) m/z (% rel. Int.), 482.00(M^+^+2, 68.5%), 481.01 (M^+^+1, 21.7%), 480.01 (M+, 100%). **Anal.** Cacld for C19H14Cl2N4O5S: C, 47.41; H, 2.93; N, 11.64; Found: C, 47.61; H, 3.06; N, 11.45.

### Ethyl 2-[N-(4-(2-methyl-4-nitroanilino)-3-nitrobenzamido)]thiazole-4-carboxylate (9d)

The titled compound was separated as greenish crystals (55%); m.p. 194–196 °C; ^**1**^**H NMR (400 MHz, DMSO-d6)** δ 10.34 (s, 1H, NH), 8.18 (s, 1H, thiazole), 8.05 (d, *J* = 8.6 Hz, 1H, ArH), 7.86 (d, *J* = 7.6 Hz 1H, ArH), 7.83 (d, *J* = 7.7 Hz, 1H, ArH), 7.78 (d, *J* = 8.8 Hz, 1H, ArH), 7.64 (s, 1H, ArH), 7.61 (s, 1H, ArH), 4.28 (q, 2H, OCH2), 3.04 (t, 3H, CH3), 2.11 (s, 3H, CH3).** MS** (Mwt= 471.44) m/z (% rel. Int.), 471.08 (M+, 100%). **Anal.** Cacld for C20H17N5O7S: C, 50.95; H, 3.63; N, 14.86; Found: C, 50.65; H, 3.93; N, 14.63.

### Ethyl 2-[N-(4-cyclohexylamino-3-nitrobenzamido)]thiazole-4-carboxylate (9e)

The titled compound was separated as buff crystals (50%); m.p. 135–137 °C; ^**1**^**H NMR (400 MHz, DMSO-d6)** δ 10.47 (s, 1H, NH), 8.07 (s, 1H, thiazole), 7.94 (d, *J* = 8.4 Hz, 1H, ArH), 7.63 (s, 1H, ArH), 7.57 (d, *J* = 8.5 Hz, 1H, ArH), 4.27 (q, 2H, OCH2), 3.05 (t, 3H, CH3), 1.28 (t, 5H, cyclohexane), 1.20 (t, 6H, cyclohexane).** MS** (Mwt = 418.47) m/z (% rel. Int.), 418.13 (M^+^, 70%), 255.83 (100%). **Anal.** Cacld for C19H22N4O5S: C, 54.53; H, 5.30; N, 13.39; Found: C, 54.33; H, 5.03; N, 13.02.

### Ethyl-2-[N-(4-(4-benzhydrylpiperazin-1-yl)-3-nitrobenzamido)]thiazole-4-carboxylate (9f)

The titled compound was separated as buff crystals (25%); m.p. 145–148 °C; ^**1**^**H NMR (400 MHz, DMSO-d6)** δ 13.13 (s, 1H, NH), 8.14 (d, *J* = 8.3 Hz, 1H, ArH), 8.12 (s, 1H, thiazole), 7.95 (s, 1H, ArH), 7.63 (d, *J* = 8.2 Hz, 1H, ArH), 7.01 (s, 10H, phenyl), 4.29 (q, 2H, OCH2), 2.89 (s, 4H, piperazine), 2.73 (s, 4H, piperazine), 1.31 (t, 3H, CH3).** MS** (Mwt = 571.65) m/z (% rel. Int.), 571.19 (M+, 100%). **Anal.** Cacld for C30H29N5O5S: C, 63.03; H, 5.11; N, 12.25; Found: C, 63.25; H, 5.36; N, 12.44.

### Ethyl 2-[N-(4-(4-methylpiperazin-1-yl)-3-nitrobenzamido)]thiazole-4-carboxylate (9 g)

The titled compound was separated as offwhite crystals (55%); m.p. 120–122 °C; ^**1**^**H NMR (400 MHz, DMSO-d6)** δ 13.13 (s, 1H, NH), 8.13 (d, *J* = 8.2 Hz, 1H, ArH), 8.12 (s, 1H, thiazole), 7.95 (s, 1H, ArH), 7.63 (d, *J* = 8.3 Hz, 1H, ArH), 4.29 (q, 2H, OCH2), 2.89 (s, 4H, piperazine), 2.73 (s, 4H, piperazine), 1.71 (t, 3H, CH3), 1.30 (t, 3H, CH3).** MS** (Mwt= 419.45) m/z (% rel. Int.), 419.13 (M^+^, 80%), 299.13 (100%). **Anal.** Cacld for C18H21N5O5S: C, 51.54; H, 5.05; N, 16.70; Found: C, 51.75; H, 5.25; N, 16.95.

### Ethyl 2-[N-(3-nitro-4-(piperid-1-yl)benzamido)]thiazole-4-carboxylate (9h)

The titled compound was separated as offwhite crystals (32%); m.p. 180–182 °C; ^**1**^**H NMR (400 MHz, DMSO-d6)** δ 10.41 (s, 1H, NH), 8.05 (s, 1H, thiazole), 7.92 (d, *J* = 8.2 Hz, 1H, ArH), 7.57 (s, 1H, ArH), 7.55 (d, *J* = 8.4 Hz, 1H, ArH), 4.24 (q, 2H, OCH2), 3.05 (t, 3H, CH3), 1.28–1.25 (m, 10H, piperidine). **MS** (Mwt = 404.44) m/z (% rel. Int.), 404.12 (M+, 100%). **Anal.** Cacld for C18H20N4O5S: C, 53.45; H, 4.98; N, 13.85; Found: C, 53.62; H, 5.10; N, 13.63.

### 2-[N-(4-Substituted-3-nitrobenzamido)]benzo[*d*]oxazole-5-carboxylic acid (10c,d,e)

#### General procedure

To a solution of LiOH.H2O (0.579 g, 13 mmol) in Ethanol (50%, 70 mL), the respective carboxylate compound **(8c, d, e)** (6.9 mmol) was added. The reaction mixture was heated under reflux for 24 h. The resulting solution was allowed to cool to room temperature then added to 10% HCl/ice. The resulting solid was filtered and washed with water to give the corresponding acids **(10c, d, e)**, yield 60–70%. The titled compounds were used in the next step directly without further purification.

### 2-[N-(4-Substituted-3-nitrobenzamido)]thiazole-4-carboxylic acid (11a,c,d,e)

#### General procedure

To a solution of LiOH.H2O (0.579 g, 13 mmol) in Ethanol (50%, 70 mL), the respective carboxylate compound **(9a, c, d, e)** (6.9 mmol) was added and we continued as mentioned in synthesis of compounds **(10c, d, e).** The titled compounds **(11a, c, d, e)** were obtained as solid powder, yield 60–70%. They were used in the next step directly without further purification.

### 5-(4-Benzhydrylpiperazin-1-oyl)-2-[N-(4-substituted-nitrobenzamido)]benzo[*d*]oxazole (12c,d,e)

#### General procedure

A solution of the respective derivative **(10c,d,e)** (1 mmol), 1-benzhydrylpiperazine (0.25 g, 1 mmol), TBTU (0.15 g, 1.2 mmol) and DMAP (0.32 g, 1 mmol) in dry DMF (15 mL) was allowed to stir under nitrogen at room temperature for 24–48 h. The completion of the reactions was confirmed using TLC. The resulting solution was poured on ice−cold water giving the titled compounds **(12c,d,e)**, yield 60–70%. They were purified by column chromatography using hexane: ethyl acetate gradient elution (9:1–1:1) and confirmed by 1H NMR spectroscopy.

### 5-(4-Benzhydrylpiperazin-1-oyl)-2-[N-(4-(3,4-dichloroanilino)-3-nitrobenzamido)] benzo[*d*]-oxazole (12c)

The titled compound was separated as brown crystals (67%); m.p. 177–180 °C; ^**1**^**H NMR (400 MHz, DMSO-d6)** δ 10.57 (s, 1H, NH), 8.14 (s, 1H, ArH), 7.99 (s, 1H, ArH), 7.97 (dd, *J* = 7.5 Hz, 2H, ArH), 7.74 (s, 1H, ArH), 7.63 (dd, *J* = 7.6 Hz, 2H, ArH), 7.34–7.27 (m, 10H, phenyl), 6.64 (dd,* J* = 7.8 Hz, 2H, ArH), 4.50 (s, 1H, CH), 2.97 (s, 8H, piperazine). **MS** (Mwt= 721.59) m/z (% rel. Int.), 722.16(M^+^+2, 63.9%), 721.17 (M++1, 41.6%), 720.17 (M^+^, 100%). **Anal.** Cacld for C38H30Cl2N6O5: C, 63.25; H, 4.19; N, 11.65; Found: C, 63.46; H, 3.96; N, 11.45.

### 5-(4-Benzhydrylpiperazin-1-oyl)-2-[N-(4−(2-methyl-4-nitroanilino)-3-nitrobenzamido)] benzo-[*d*]oxazole (12d)

The titled compound was separated as greenish crystals (65%); m.p. 195–197 °C; ^**1**^**H NMR (400 MHz, DMSO-d6)** δ 10.21 (s, 1H, NH), 8.19 (s, 1H, ArH), 8.11 (d, *J* = 8.5 Hz, 1H, ArH), 8.01 (d, *J* = 8.2 Hz, 1H, ArH), 7.95 (s, 1H, ArH), 7.78 (d, *J* = 8.9 Hz, 1H, ArH), 7.63 (d, *J* = 8.1 Hz, 1H, ArH), 7.47–7.15 (m, 3H, ArH), 7.04 (s, 10H, phenyl), 4.48 (s, 1H, CH), 2.89 (s, 4H, piperazine), 2.73 (s, 4H, piperazine), 2.39 (s, 3H, CH3). **MS** (Mwt= 711.72) m/z (% rel. Int.), 711.24 (M+, 66%), 530.43 (100%). **Anal.** Cacld for C39H33N7O7: C, 65.81; H, 4.67; N, 13.78; Found: C, 66.03; H, 4.89; N, 13.55.

### 5-(4-Benzhydrylpiperazin-1-oyl)-2-[N-(4-cyclohexylamino-3-nitrobenzamido)]benzo[*d*]-oxazole (12e)

The titled compound was separated as buff crystals (62%); m.p. 187–190 °C; ^**1**^**H NMR (400 MHz, DMSO-d6)** δ 10.52 (s, 1H, NH), 7.92 (dd, 2H, ArH), 7.73 (s, 1H, ArH), 7.68 (dd, 2H, ArH), 7.65 (s, 1H, ArH), 7.43–7.18 (m, 10H, phenyl), 4.42 (s, 1H, CH), 3.09–2.97 (m, 8H, piperazine), 1.20–1.16 (m, 11H, cyclohexane). **MS** (Mwt = 658.75) m/z (% rel. Int.), 658.29 (M^+^, 100%). **Anal.** Cacld for C38H38N6O5: C, 69.28; H, 5.81; N, 12.67; Found: C, 69.06; H, 5.60; N, 12.43.

### 4-(4-Benzhydrylpiperazin-1-oyl)-2-[N-(4-substituted-3-nitrobenzamido)]thiazole (13a,c,d,e)

#### General procedure

A solution of the respective acid derivative **(11a,c,d,e)** (1 mmol), 1−benzhydrylpiperazine (0.25 g, 1 mmol), TBTU (0.15 g, 1.2 mmol) and DMAP (0.32 g, 1 mmol) in dry DMF (15 mL) was allowed to stir under nitrogen at room temperature for 24–48 h. The completion of the reactions was confirmed using TLC. The resulting solutions were poured on ice−cold water giving the titled compounds **(13a,c,d,e)**, yield 60–70%. They were purified by column chromatography using hexane: ethyl acetate gradient elution (9:1–1:1) and confirmed by 1H NMR spectroscopy.

### 4-(4-Benzhydrylpiperazin-1-oyl)-2-[N-(4-(phenethylamino)-3-nitrobenzamido)]thiazole (13a)

The titled compound was separated as white crystals (28%); m.p. 232–234 °C; ^**1**^**H NMR (400 MHz, DMSO-d6)** δ 8.13 (s, 1H, NH), 7.74 (s, 1H, thiazole), 7.42 (m, 10H, phenyl), 7.28 (m, 5H, phenyl), 7.17 (m, 3H, ArH), 4.25 (s, 1H, CH), 2.75 (s, 4H, piperazine), 2.25 (s, 4H, piperazine), 1.30 (t, 4H, CH2CH2). **MS** (Mwt= 646.76) m/z (% rel. Int.), 646.24 (M^+^, 88%), 167.20 (100%). **Anal.** Cacld for C36H34N6O4S: C, 66.85; H, 5.30; N, 12.99; Found: C, 66.66; H, 5.54; N, 13.14.

### 4-(4-Benzhydrylpiperazin-1-oyl)-2-[N-(4-(3,4-dichloroanilino)-3-nitrobenzamido)] thiazole (13c)

The titled compound was separated as brown crystals (37%); m.p. 225–227 °C; ^**1**^**H NMR (400 MHz, DMSO-d6)** δ 10.54 (s, 1H, NH), 8.06 (s, 2H, ArH), 7.91 (s, 1H, thiazole), 7.15–7.13 (m, 10H, phenyl), 6.86 (d,* J* = 8.1 Hz, 1H, ArH), 6.74 (d, *J* = 8.3 Hz, 1H, ArH), 6.52 (dd, 2H, ArH), 4.31 (s, 1H, CH), 2.51 (s, 4H, piperazine), 1.93 (s, 4H, piperazine). **MS** (Mwt= 687.59) m/z (% rel. Int.), 688.12(M^+^+2, 69.8%), 687.13 (M^+^+1, 40.6%), 686.13 (M^+^, 100%). **Anal.** Cacld for C34H28Cl2N6O4S: C, 59.39; H, 4.10; N, 10.13; Found: C, 59.19; H, 3.96; N, 10.36.

### 4-(4-Benzhydrylpiperazin-1-oyl)-2-[N-(4-(2−methyl-4-nitroanilino)-3-nitrobenzamido)]-thiazole (13d)

The titled compound was separated as yellow crystals (29%); m.p. 207–209 °C; ^**1**^**H NMR (400 MHz, DMSO-d6)** δ 10.54 (s, 1H, NH), 7.95 (s, 1H, thiazole), 7.88 (dd, 2H, ArH), 7.85 (s, 1H, ArH), 7.22–7.15 (m, 10H, phenyl), 6.66 (dd, 2H, ArH), 6.48 (s, 1H, ArH), 4.25 (s, 1H, CH), 2.88 (s, 4H, piperazine), 2.73 (s, 4H, piperazine), 2.12 (s, 3H, CH3). **MS** (Mwt= 766.73) m/z (% rel. Int.), 677.21 (M^+^, 90%), 499.39 (100%). **Anal.** Cacld for C35H31N7O6S: C, 62.03; H, 4.61; N, 14.47; Found: C, 62.25; H, 4.81; N, 14.23.

### 4-(4-Benzhydrylpiperazin-1-oyl)-2-[N-(4-cyclohexylamino-3-nitrobenzamido)]thiazole (13e)

The titled compound was separated as offwhite crystals (21%); m.p. 174–176 °C; ^**1**^**H NMR (400 MHz, DMSO-d6)** δ 10.52 (s, 1H, NH), 9.63 (s, 1H, thiazole), 7.94 (d, *J* = 8.1 Hz, 1H, ArH), 7.66 (s, 1H, ArH), 7.56 (d, *J* = 8.0 Hz, 1H, ArH), 7.47–7.25 (m, 10H, phenyl), 4.20 (s, 1H, CH), 3.33–3.02 (m, 8H, piperazine), 1.44–0.99 (m, 11H, cyclohexane).** MS** (Mwt = 624.75) m/z (% rel. Int.), 624.25 (M^+^, 80%), 455.24 (100%). **Anal.** Cacld for C34H36N6O4S: C, 65.36; H, 5.81; N, 13.45; Found: C, 65.15; H, 5.63; N, 13.23.

## Biological evaluation

### In vitro anti−proliferative activity against NCI 60 cell line panel

The NCI in vitro anticancer screening is the evaluation of the selected compounds against the full NCI 60 cell lines panel representing leukemia, Non−Small Cell Lung Cancer, melanoma, colon cancer, CNS cancer, breast cancer, ovarian cancer, renal cancer and prostate cancer at a single dose of 10 µM. The output from the single dose screen is reported as a mean graph [21]. Assay protocol and data analysis are in the supplementary part. Inhibitory concentration 50% (IC_50_) values were determined for compounds** 8 g** and **12e**. Standard deviations were calculated using the IC_50_ values obtained from 3 independent experiments. HCT-116 cell line was purchased from Vacsera, Tissue culture unit and experiments were performed in Science way for scientific research and consultations. The MTT protocol performed is provided in the supplementary section.

### Cell cycle analysis

This assay was carried out in The Research and Development Center, Faculty of Medicine, Al−Azhar University. Materials used are Propidium Iodide Stain, 0.05% trypsin, and PBS Buffer (Phosphate−buffered Saline). Assay protocol is in the supplementary part.

### Annexin V-FITC assay

This assay was carried out in The Research and Development Center, Faculty of Medicine, Al−Azhar University. Annexin V-FITC 1X Binding Buffer and Propidium Iodide(PI) were used. The assay protocol is provided in the supplementary section.

### Detection of caspase-3 protein assay

This assay was carried out in The Research and Development Center, Faculty of Medicine, Al−Azhar University. Materials used were Caspase-3 (active): Catalog # KHO1091, Antibody Coated Wells, Caspase-3 (Active) Detection Antibody, Anti−Rabbit IgG HRP (100X), Standard Diluent Buffer. Contains 0.1% sodium azide, red dye*.* Assay protocol and data analysis are in the supplementary part.

### In vitro Bcl-2 activity

The in vitro inhibition assay for the synthesized compounds was carried out in BPS Bioscience Corporation, San Diego, CA, USA (www.bpsbioscience.com). The assay was performed by TR-FRET technology using a recombinant Bcl-2 and a peptide− ligand substrate. The TR-FRET signal from the assay is correlated with the amount of Ligand binding to Bcl-2. The % inhibition caused by the tested compounds against Bcl-2 was evaluated compared to a reference Bcl-2 inhibitor ABT-199 at a single concentration of 10 µM. Materials used are Bcl-2: Catalog # 50272, Bcl-2 binding peptide, Bcl-2 Assay Kit: Catalog #50222, and Tb-Donor and Dye labeled acceptor. Assay protocol and data analysis are in the supplementary part.

### PCR analysis and quantification of gene expression of Bax, Bcl-2, Bcl-xL

This assay was carried out in The Research and Development Center, Faculty of Medicine, Al−Azhar University. iScript^™^ One−Step RT−PCR Kit with SYBR^®^ Green was used. Primer sequences were as follows: Bcl-2 forward primer –ATCGCCCTGTGGATGACTGAGT− and reverse primer −GCCAGGAGAAATCAAACAGAGGC−; BAX forward primer −TCAGGATGCGTCCACCAAGAAG− and reverse primer 5′−TGTGTCCACGGCGGCAATCATC−; Bcl-xL forward primer −GCCACTTACCTGAATGACCACC − and reverse primer −AACCAGCGGTTGAAGCGTTCCT −; reference housekeeping gene used was GAPDH with forwarding primer −CATCACTGCCACCCAGAAGACTG− and reverse primer −ATGCCAGTGAGCTTCCCGTTCAG−. Assay protocol and data analysis are in the supplementary part.

### Effect of representative target compounds on normal human cell lines

This assay was performed at the cell culture unit in center for drug discovery research and development at faculty of pharmacy, Ain shams university and the assay protocol is provided in the Additional file 1.

## Results and discussion

### Chemistry

**Scheme 1:**
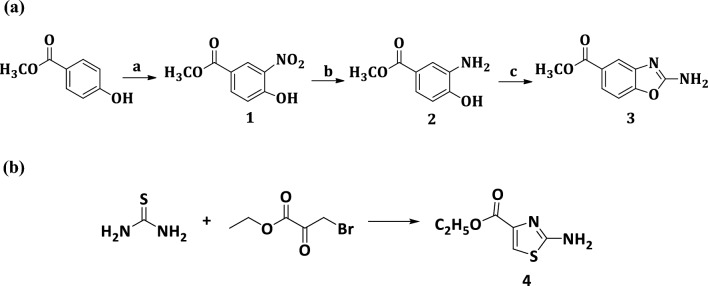
**(a)** Synthesis of methyl 2-aminobenzo[*d*]oxazole-5-carboxylate (3), Reagents and conditions: **a** Al(NO_3_)_3_, Acetic anhydride, gl. AcOH, rt, 1.5 h, 85% **b** Na dithionite, Acetone, 0.5N NaOH, reflux, 1 h, 70% **c** CNBr, MeOH, rt, 3–4 h, 70%. **(b)**  Synthesis of ethyl 2-aminothiazole-4-carboxylate (4), Reagents and conditions: EtOH, reflux, 24 h, 72%

**Scheme 2:**
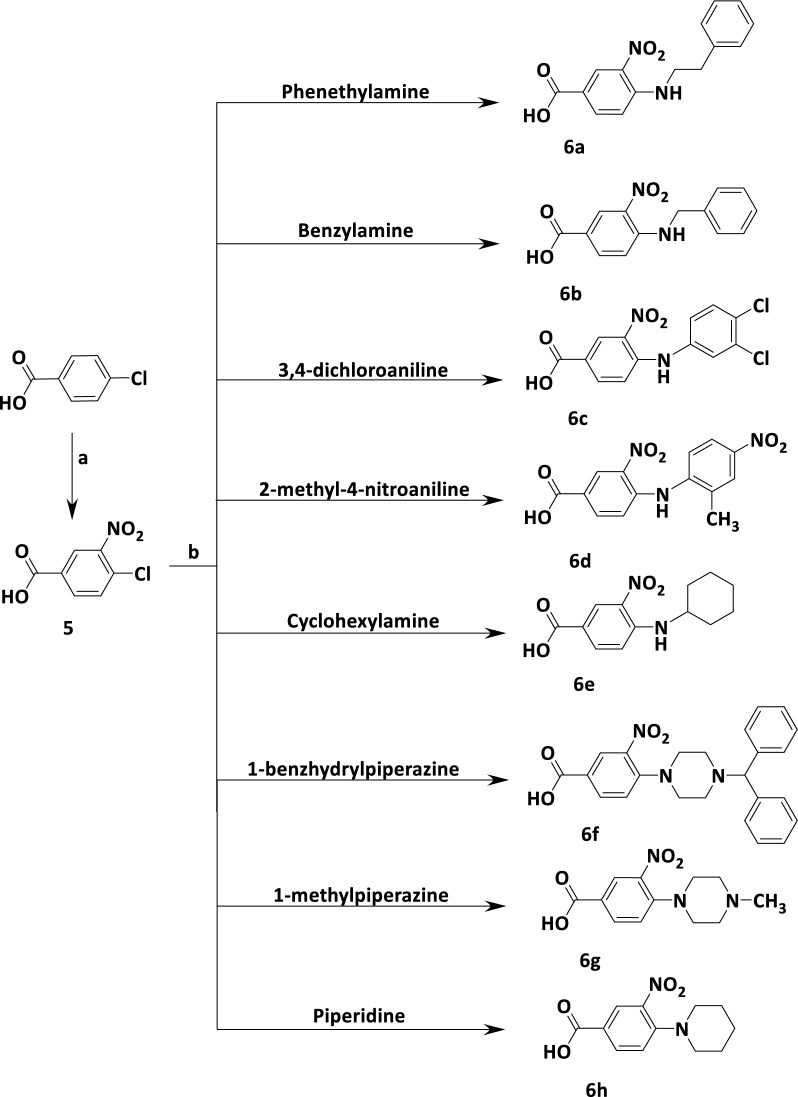
Synthesis of 4-substituted-3-nitrobenzoic acid (6a−h), Reagents and conditions: **a** NaNO_3_, H_2_SO_4_, 0 °C then rt, 24 h, 90% **b** K_2_CO_3_, DMF, rt, 8–24 h, 55–65%

**Scheme 3:**
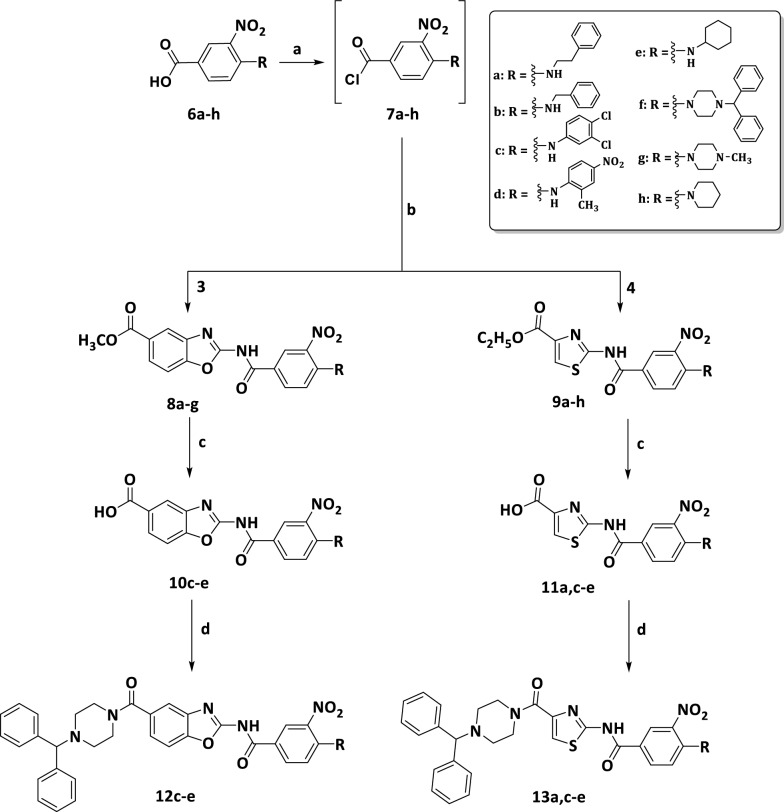
Synthesis of targeted compounds (12c−e) and (13a,c−e), Reagents and conditions: **a** SOCl_2_, DCM, reflux, 7 h, 50–60% **b** TEA, DCM, rt, 24–48 h, 20–60% **c** LiOH.H_2_O, EtOH, reflux, 24 h, 60–70% **d** 1−benzhydrylpiperazine, TBTU, DMAP, DMF, under N_2_, rt, 24–48 h, 20–40%

The synthetic pathways for preparation of the target compounds were depicted in Schemes 1, 2 and 3. Final compounds incorporating substituted amides were obtained utilizing the corresponding intermediates **3** and **4**, which were synthesized according to the routes outlined in Scheme 1a, b. The nitration reaction of methyl 4-hydroxybenzoate with Al(NO_3_)_3_ results in the formation of methyl 4-hydroxy-3-nitrobenzoate **(1)** [20]. Using sodium dithionite, the nitro group of compound **1** was reduced into amino group to give methyl 3-amino-4-hydroxybenzoate **(2)** [12]. The methyl 2-aminobenzo[*d*]oxazole-5-carboxylate **(3)** [13] was prepared by reacting compound **2** with cyanogen bromide aqueous suspension (Scheme 1a). On the other hand, ethyl 2-aminothiazole-4-carboxylate **(4)** [14] was prepared by condensation of thiourea with ethyl bromopyruvate (Scheme 1b).

The synthesis of 4-substituted-3-nitrobenzoic acid derivatives **(6a−h)** was obtained in two steps. The first step was the nitration of 4-chorobenzoic acid using a mixture of concentrated nitric acid and sulfuric acid as nitrating reagent to prepare 4-chloro-3-nitrobenzoic acid **(5)** [15]. The second step was nucleophilic substitution of activated compound **5** with different primary and secondary amines using K_2_CO_3_ as a base and DMF as a solvent to obtain the 4-substituted-3-nitrobenzoic acids **(6a−h)** (Scheme 2). The resulted compounds **(6a−h)** were then activated through reaction with thionyl chloride [22] to afford 4-substituted-3-nitrobenzoyl chloride **(7a−h) **(Scheme 3). The activated acid chlorides **(7a−h)** were immediately reacted with either compound **3** or compound **4** in presence of TEA to give the amide benzoxazole−based derivatives **(8a−g)** and the amide thiazole−based derivatives **(9a−h)** respectively [23, 24].

The ester moiety of compounds **8c−e** and **9a,c−e** were then hydrolyzed to their corresponding acids **(10c−e)** and **(11a,c−e)** using LiOH.H2O under overnight reflux. [25] They were used directly in the next step to obtain the final amide compounds **(12c−e)** and **(13a,c−e)** through coupling with 1−benzhydrylpiperazine using TBTU as coupling reagent under N2 [26] (Scheme 3). The 1HNMR signals were consistent with protons of the targeted compounds **(12c-e)** and **(13a,c-e).**

## Biological evaluation

### In vitro anti−proliferative activity against NCI 60-cell line

This assay was performed for all the final compounds **(8a-g, 9a-h, 12c-e, 13a, c-e)** by the Developmental Therapeutics Program (DTP) of the National Cancer Institute (NCI), division of cancer treatment and diagnosis, NIH, Bethesda, Maryland, USA (www.dtp.nci.nih.gov). The operation of this screen utilizes 60 different human tumor cell lines [21]. Compounds **8a-g, 9a-h, 12c-e, 13a,c-e** were tested at initial single dose 10 µM inhibition percent assay on the full NCI 60 cell panel. The results are expressed as cell growth percent for each compound and reported as a mean graph of the percent growth of the treated cells compared to the untreated control cells. The obtained results have been illustrated in the supplementary material. From analysis of these results, the following observations could be outlined: Nine compounds **(8c, 8g, 9a, 9f, 9g, 12d, 12e, 13a and 13c)** showed more than 40% growth inhibition to the leukemic K-562 cell line. The leukemic CCRF−CEM cell line was inhibited by seven of the synthesized compounds **(8a, 8g, 9b, 12d, 12e, 13c and 13d)** with 39–81% cell growth inhibition range. While the RPMI-8226 cell line was inhibited by compounds **(8g, 9a, 9f, 12d, 12e, 13a and 13c)** with 30–73% cell growth inhibition range.

Compound **8g** showed the highest cell growth inhibition to the 60-cell line panel with mean growth inhibition of 45.13%. It exhibited broad spectrum and good anti−proliferative activity against several cell lines such as the leukemic CCRF−CEM and the colon cancer HCT-15, HCT-116 cell lines with growth inhibition 81, 67.52 and 66.06%, respectively.

Compound **12e** with mean growth inhibition of 42.29% exhibited anti−proliferative activity against several cell lines such as the leukemic CCRF−CEM and the colon cancer HCT-15, HCT-116 cell lines with growth inhibition 71.71, 64.91 and 59.11%, respectively.

Compound **13d** with mean growth inhibition of 29.25% exhibited anti−proliferative activity against several cell lines: the NSCLC NCI-H460 and the colon cancer HCT-15, HCT-116 cell lines with growth inhibition 61.85, 44.68 and 43.44%, respectively.

These three compounds **(8g, 12e, 13d)** are representative to different series, and they showed the best cell growth inhibition percent to the available HCT-116 cell line. Accordingly, IC50 values were determined for compounds **8g** and **12e** to be 89.91±2.39 and 112.58±2.55 µg/ml,respectively.

### Cell cycle analysis

There is a direct link between apoptosis and the cell cycle as mitosis and apoptosis display very similar morphological features. Apoptosis is actively linked to the G2/M phases [27] through the apoptotic inducer p53 which may induce a G2 arrest through its transactivation function. For example, it activates the pro−apoptotic proteins, Bax, and plays an important role in activation of caspase−dependent apoptosis [28].

**Fig. 4:**
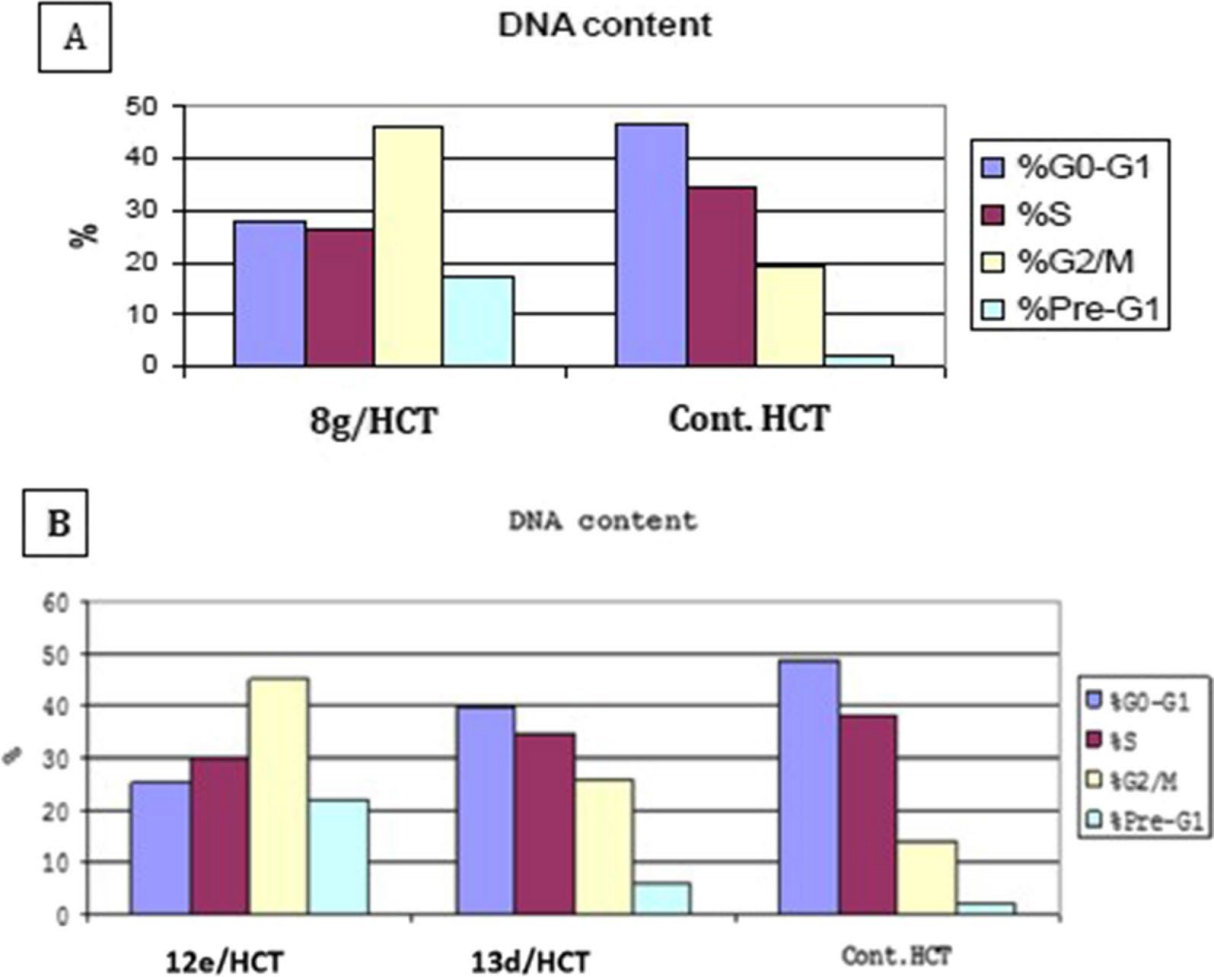
Bar chart representing the effect of: **A** compound 8g, **B** compounds 12e, 13d on the cell cycle phases of HCT-116 cells

**Fig. 5:**
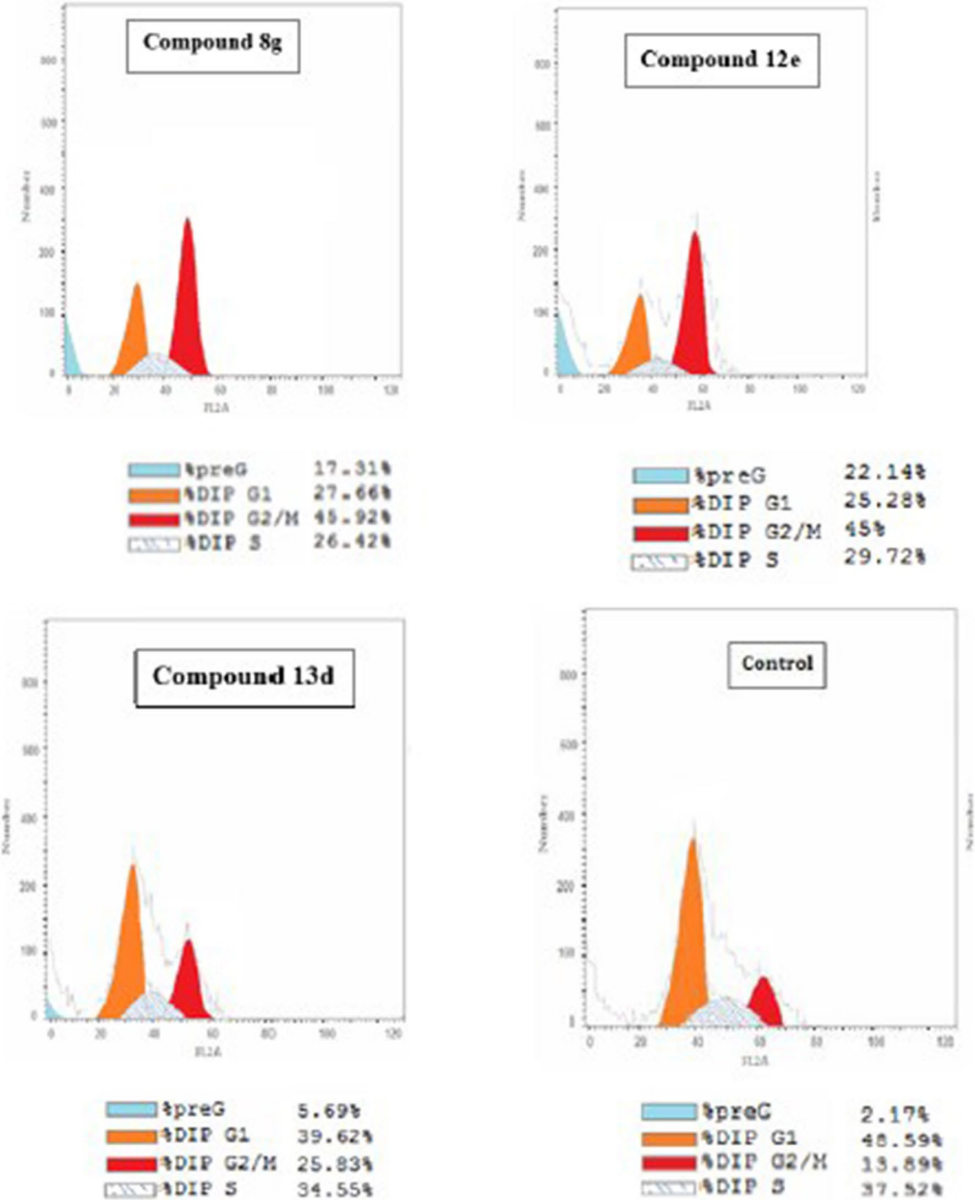
Graphs representing cell count in each cell cycle phase upon treatment with compounds 8g, 12e, 13d vs. the untreated HCT-116 cells

To better elucidate the mechanism of action of the three most active synthesized compounds **(8g, 12e, 13d)**, they were tested for their effect on altering cell cycle in comparison with untreated HCT-116 cell line as a control. The results on the cell cycle phases, induction of apoptosis, and cell count in each cell cycle phase of HCT-116 cell line upon treatment with compounds **8g, 12e, 13d** are illustrated in Figs.  4 and 5.

As shown in the bar charts: The representative compounds **(8g, 12e, 13d)** caused PreG1 apoptosis and arrested the cells in G2/M phase.

From the previous graphs, it was observed that there is an increase in the number of cells treated with compounds **8g, 12e, 13d** that entered PreG1 phase from 2.17 to 17.31, 22.14 and 5.69%, respectively in comparison to the control cells in addition to increase in ratio of the cells in G2/M phases from 13.89 to 45.92, 45.0 and 25.83, respectively. On the other hand, a decrease in the ratio of the cells in G1 phase from 48.59 to 27.66, 25.28 and 39.62%, respectively and also a decrease in the ratio of the cells in S phase from 37.52 to 26.42, 29.72 and 34.55%, respectively were detected. Therefore, compounds **8g, 12e, 13d** induced apoptosis and complete cell growth arrest occurred at G2/M phase.

### Annexin V−FITC assay

**Fig. 6:**
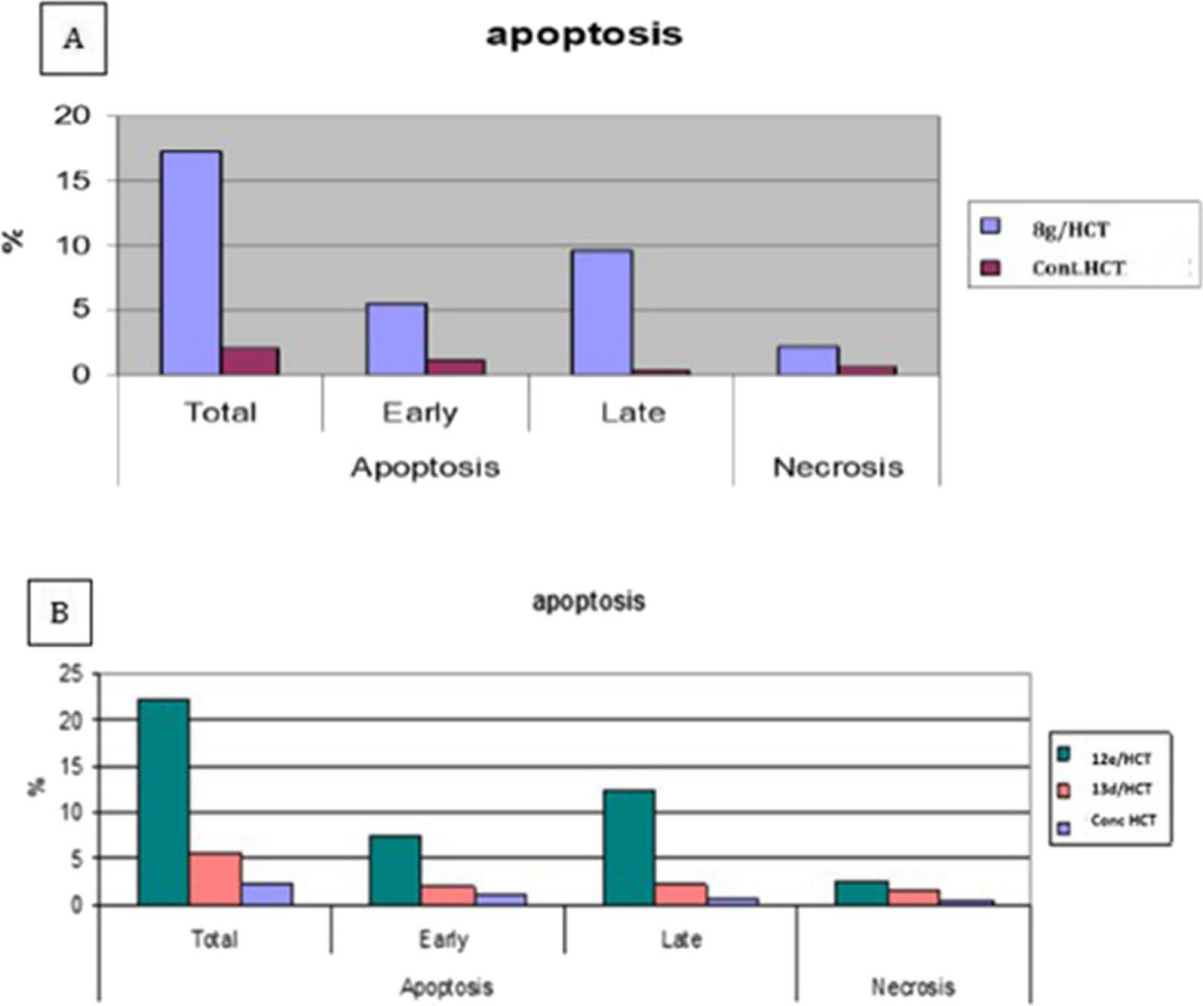
Bar chart representing the effect of: **A**. compound 8g, **B**. compounds 12e, 13d on induction of apoptosis of HCT-116 cells

**Fig. 7:**
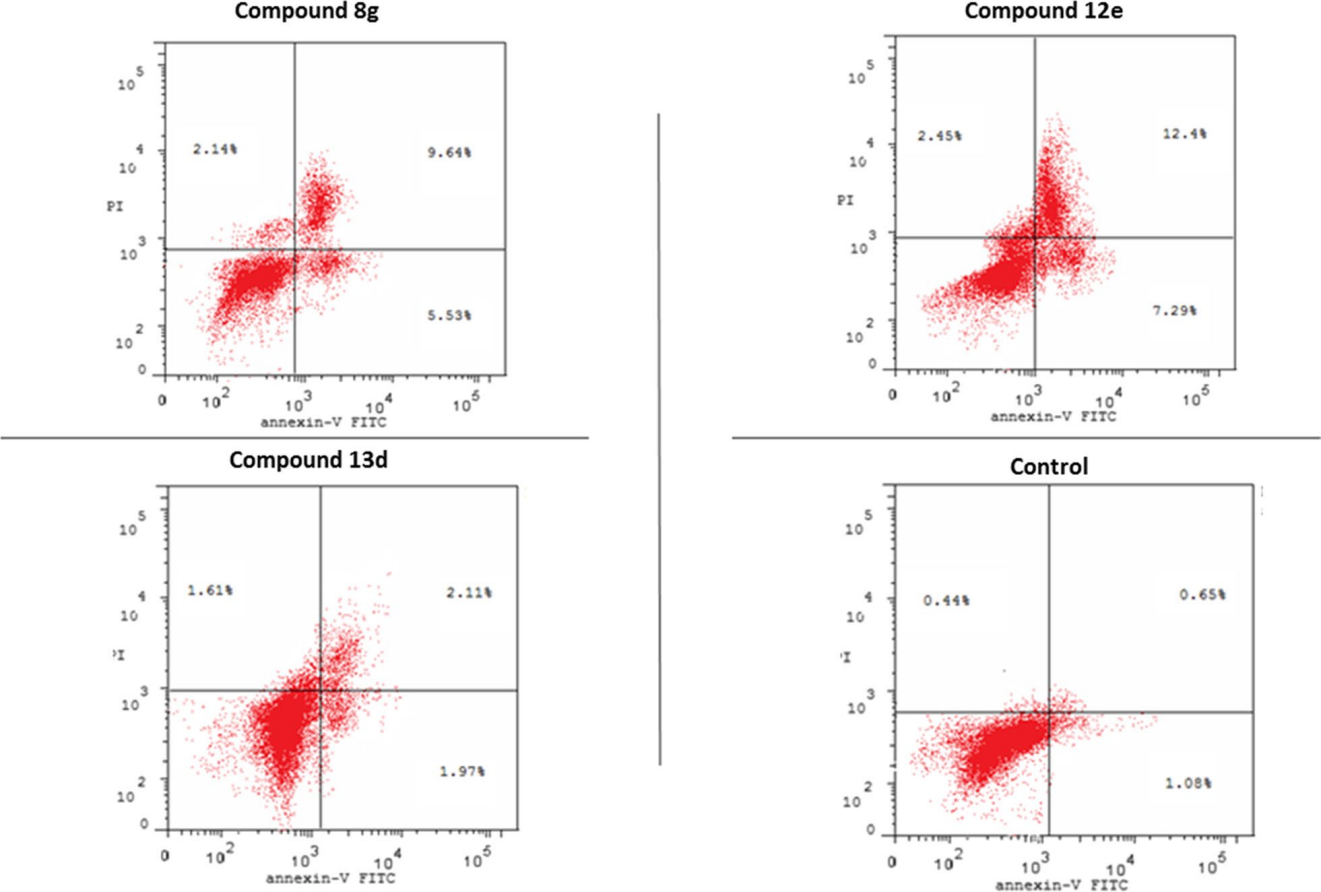
Dot plots representing the effect of: compounds 8g, 12e and 13d on apoptosis progression of HCT-116 cells

To further investigate the effects of compounds **8g, 12e, 13d** on apoptosis progression, annexin V-FITC assay was carried out. As illustrated in the bar charts (Fig. 6) and the dot plots (Fig. 7), the tested compounds declared increase in both early and late apoptosis of the treated HCT-116 cells to be 5.53% and 9.64% for compound **8g**, 7.29 and 12.4% for compound **12e** and 1.97 and 2.11% for compound **13d**. This resulted in a significant increase in the total apoptosis to be 15.17, 19.69 and 4.08% for the former compounds, respectively compared to the untreated HCT-116 cells which showed 1.73% total apoptosis (1.08% for early apoptosis and 0.65% for late apoptosis).

### Detection of caspase-3 protein assay

**Fig. 8:**
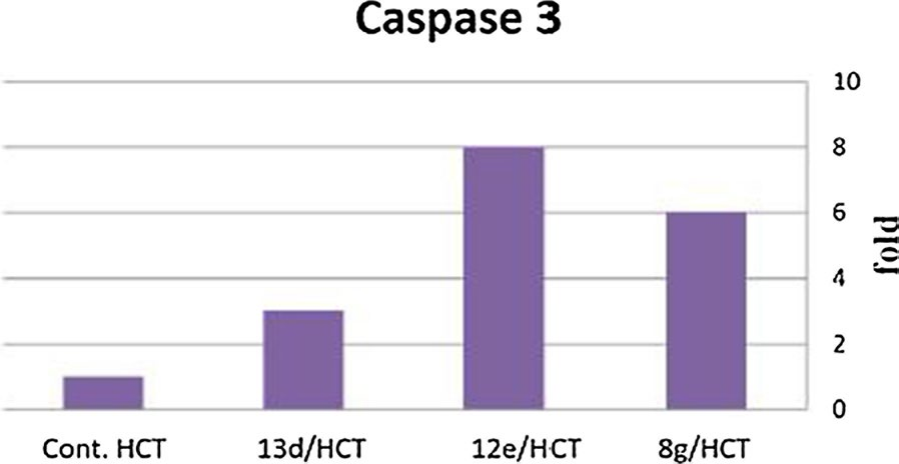
The effect of compounds 8g, 12e, 13don caspase−3 level in HCT-116 cells

Caspase-3 is a cysteine protease with aspartic specificity and a well−characterized effector on apoptosis. It is synthesized as inactive proenzyme, where upon cleavage at Asp175/Ser176, is converted to the active enzyme. The best recognized biochemical hallmark of apoptosis is the activation of caspases. The purpose of this assay is to detect and quantify the level of human active caspase-3 protein when cleaved at Asp175/Ser176 using ELISA kits. Compounds **8g, 12e, 13d** were tested for their effect on altering caspase-3 expression level on HCT-116 human cell line in comparison with the same untreated ones. The three compounds showed a massive increase (6 folds, 8 folds and 3 folds, respectively) in the caspase-3 levels as shown in Fig. 8, which confirms that the synthesized compounds can induce apoptosis through a caspase−dependent pathway.

### In vitro Bcl-2 inhibitory activity assay

The *in−vitro* Bcl-2 inhibition assay was performed at BPS Bioscience Corporation, San Diego, CA, USA. The purpose of this study is to determine the effect of the three compounds **(8g, 12e, 13d)** which showed the best anti proliferative activity against NCI 60 cell lines on human Bcl-2. It was conducted at a single concentration of 10 µM compared to a reference Bcl-2 inhibitor ABT-199 (at 100 nM).

Compounds **8g, 12e, 13d** showed significant Bcl-2 inhibitory activity with 69.2% inhibition achieved by compound **12e**. Hence, these compounds are good inhibitors of Bcl-2 anti−apoptotic protein as expected (Table 1).

**Table 1:** Percent inhibition of Bcl-2 achieved by the targeted compounds (8g, 12e, 13d) at 10 µM

Compound ID	Bcl-2 inhibition%
8g	60.2
12e	69.2
13d	50.0
ABT−199 (at 100 nM)	92.0

### PCR analysis and quantification of gene expression of Bax, Bcl-2, Bcl-xL

**Fig. 9:**
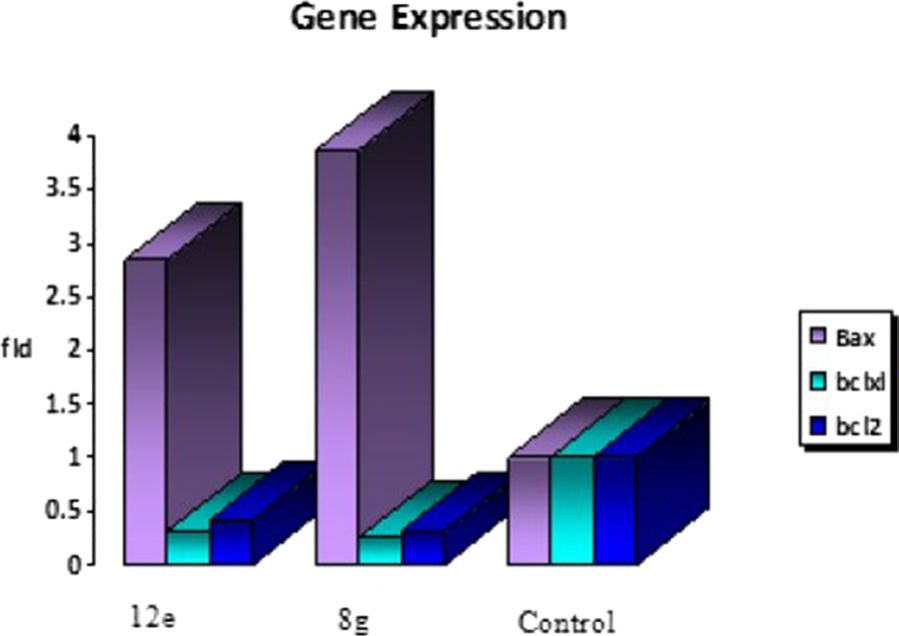
The fold changes of Bax, Bcl-xL and Bcl2 levels after treatment with compounds 8g and 12e

Bcl-2 and Bcl-xL anti−apoptotic proteins along with Bcl-2-associated X protein (Bax) as proapoptotic protein play crucial role in tumor progression by inhibition of the apoptotic pathway. The aforementioned proteins’ expression levels were determined after treating HCT-116 cell line with IC_50_ of the top two compounds **8g** and **12e.** As shown in Table 2, proapoptotic protein Bax levels were elevated 3.864 and 2.834 folds compared to the control for compounds **8g** and **12e** respectively. Meanwhile, levels of antiapoptotic proteins Bcl-2 and Bcl-xL were down−regulated by 0.31 and 0.251 folds for compound **8g** and 0.415 and 0.314 folds for compound **12e** respectively (Fig. 9). Interestingly, compounds **8g** and **12e** were found to increase the Bax/Bcl-2 ratio by around 12 and 7 folds compared to the control. These results emphasize the proapoptotic activity of the tested compounds.

**Table 2:** Effect of compounds 8 g and 12e on the expression of Bax, Bcl-xL and Bcl2 genes

Sample			RT-PCR results fold change		
Compound number	Cells	IC_50_ (ug/ml)	Bax	Bcl-xL	Bcl-2
8g	HCT-116	89	3.864	0.251	0.31
12e	HCT-116	112	2.834	0.314	0.415
Control	–	–	1	1	1

### Effect of representative target compounds on normal human cell lines

For further exploration of the safety profile of compounds **8g** and **12e**, cytotoxicity assay versus normal human fibroblast cells was performed. The IC_50_ values were determined to be 840.4 ug/ml for compound **8g** while it was not covered for compound **12e** as it was exceeding 1000 ug/ml. The results reveal that both compounds are safe and non−toxic to normal cells.

## Conclusion

Twenty two novel benzoxazole and thiazole−based compounds were designed, synthesized as Bcl-2 inhibitors. The targeted compounds were evaluated for their anti−proliferative activity against NCI 60−cell line panel. Compounds **8g, 12e, 13d** showed good to moderate anti−proliferative activity against most of the NCI cell lines with mean growth inhibition percent of 45.13, 42.29 and 29.25%, respectively. The cell growth inhibition was observed with HCT-116 cell line with values of 68.0, 59.11 and 43.44% respectively therefore IC50 values were determined for the two compounds with the greatest inhibition percent (**8g** and **12e**) to be 89.91 ± 2.39 and 112.58±2.55 µg/ml respectively. The three compounds **(8g, 12e, 13d)** were subjected to cell cycle analysis to test their effect on apoptosis and altering cell cycle. They showed PreG1 apoptosis and arrested the cells in G2/M phase with 17, 22 and 5% increase in the total apoptosis of HCT-116 cell line, respectively.

Furthermore, they were tested for their effect on altering caspase-3 expression level on HCT-116 human cell line in comparison with the same untreated ones. Compounds **8g, 12e, 13d** showed a significant increase in caspase−3 levels (6 folds, 8 folds and 3 folds, respectively) which assures that the synthesized compounds can induce apoptosis through a caspase−dependent pathway.

The *in−vitro* Bcl-2 inhibitory activity was carried on the three compounds and they showed very good inhibition of Bcl-2. The effectiveness of the most active compounds **(8g and 12e)** as apoptotic inducers was confirmed by the marked increase in Bax level and downregulation of Bcl-2 and Bcl-xL levels compared to the control.

Our study suggests that optimization of these designed compounds may lead to developing more active hits targeting different cancer types especially colorectal cancer through apoptotic induction.

### Supplementary Information

**Additional file 1: Figure S1.** Mean graph of compound (8g) produced from NCI 60 cell line screening program; color codes are given for each cell line. **Table S1.** Cell growth inhibition percentage of NCI 60 cancer cell lines exhibited by investigated final compounds. **Table S2.** Determination of sample cytotoxicity on HCT-116 cells (MTT protocol). **Figure S2.** Effect of 8g and 12e on HCT-116 cells at different concentrations. **Figure S3.** Effect of 12e on HCT-116 cells at different concentrations.

## Data Availability

All the data generated during this study are incorporated in this published article and Additional file 1.
