# Supplementary material for: Targeting apoptosis; design, synthesis and biological evaluation of new benzoxazole and thiazole based derivatives
Source: BMC Chem. 2024 Jan 3;18(1):1. doi: 10.1186/s13065-023-01101-2 (PMC10765682; doi:10.1186/s13065-023-01101-2)
Supplement: Supplementary file 1 — Additional file 1: Figure S1. Mean graph of compound (8g) produced from NCI 60 cell line screening program; color codes are given for each cell line. Table S1. Cell growth inhibition percentage of NCI 60 cancer cell lines exhibited by investigated final compounds. Table S2. Determination of sample cytotoxicity on HCT-116 cells (MTT protocol). Figure S2. Effect of 8g and 12e on HCT-116 cells at different concentrations. Figure S3. Effect of 12e on HCT-116 cells at different concentrations. [file 13065_2023_1101_MOESM1_ESM.docx]

**Targeting apoptosis; design, synthesis and biological evaluation of new benzoxazole and thiazole based derivatives**

**Chemistry:**

**^1^H NMR and mass spectra of the target compounds 8a-g, 9a-h, 12c-e, 13a,c-e and IR of 11a.**

**Methyl 2-[N-(3-nitro-4-phenethylaminobenzamido)]benzo[*d*]oxazole-5-carboxylate (8a)**

**
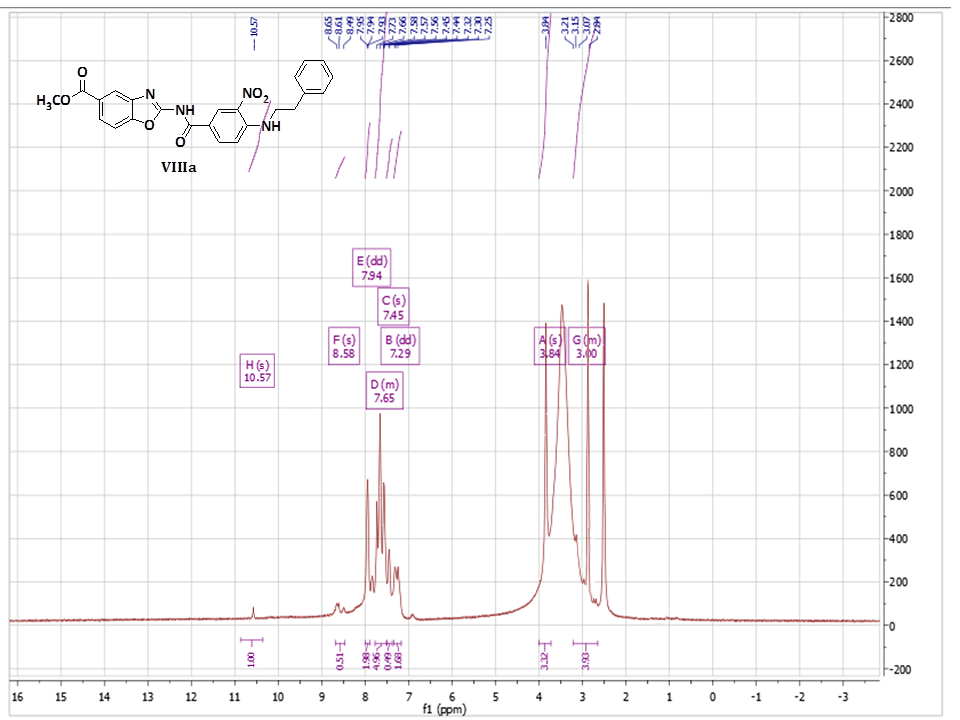
**

**Methyl 2-[N-(4-benzylamino-3-nitrobenzamido)]benzo[*d*]oxazole-5-carboxylate (8b)**

**
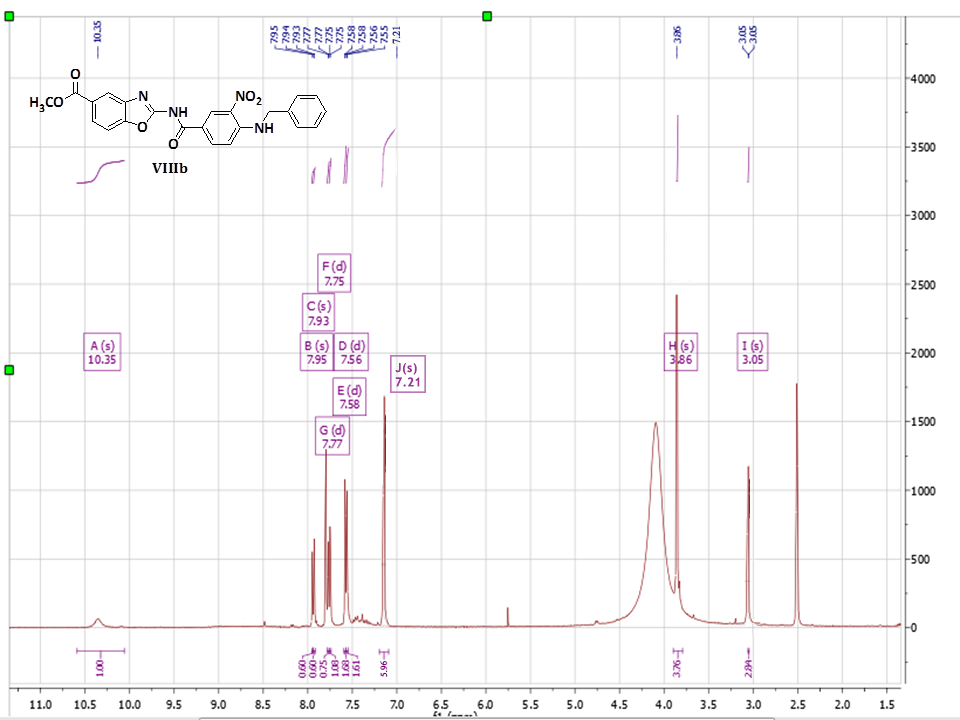
**

**Methyl 2-[N-(4-(3,4-dichloroanilino)-3-nitrobenzamido)]benzo[*d*]oxazole-5-carboxylate (8c)**


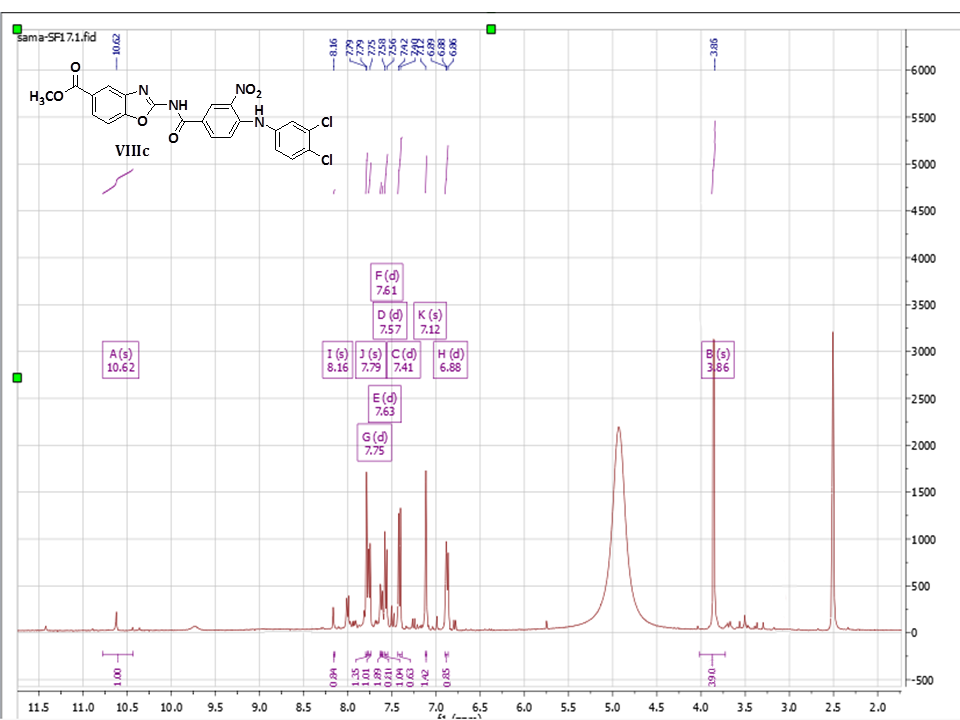

**Methyl 2-[N-(4-(2-methyl-4-nitroanilino)-3-nitrobenzamido)]benzo[*d*]oxazole-5-carboxylate (8d)**

**
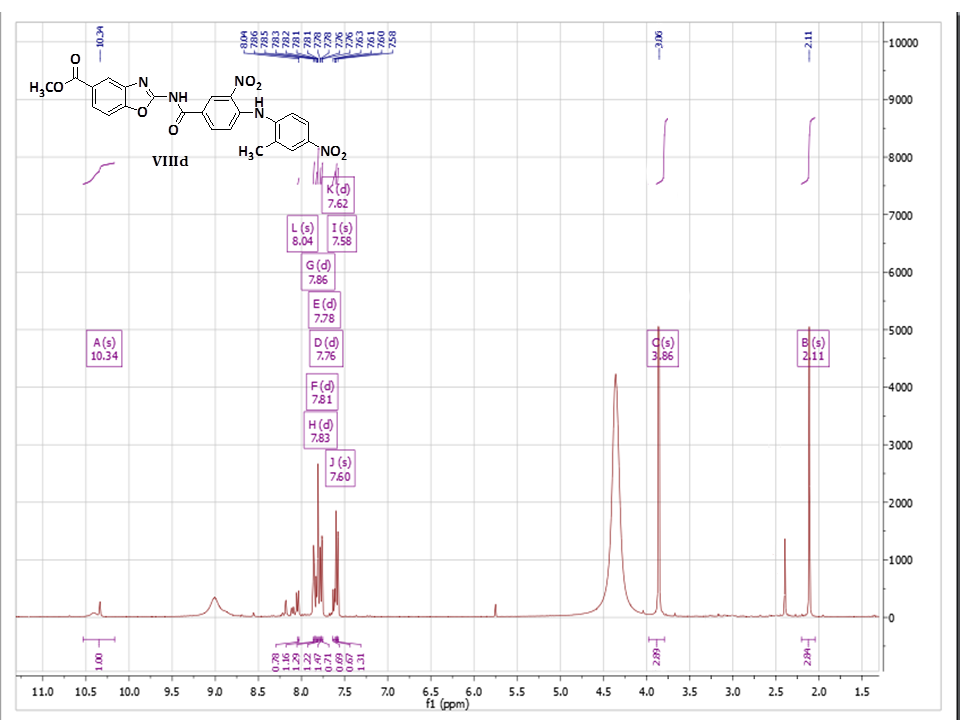
**

**Methyl 2-[N-(4-cyclohexylamino-3-nitrobenzamido)]benzo[*d*]oxazole-5-carboxylate (8e)**

**
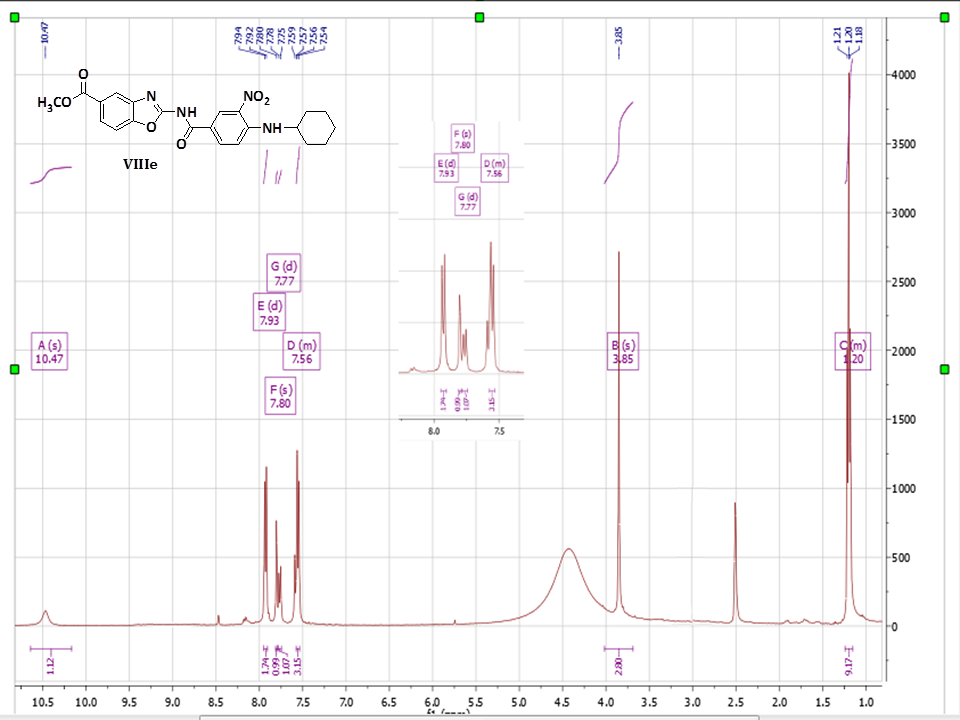
**

**Methyl 2-[N-(4-(4-benzhydrylpiperazin-1-yl)-3-nitrobenzamido)]benzo[*d*]oxazole-5-carboxylate (8f)**

**
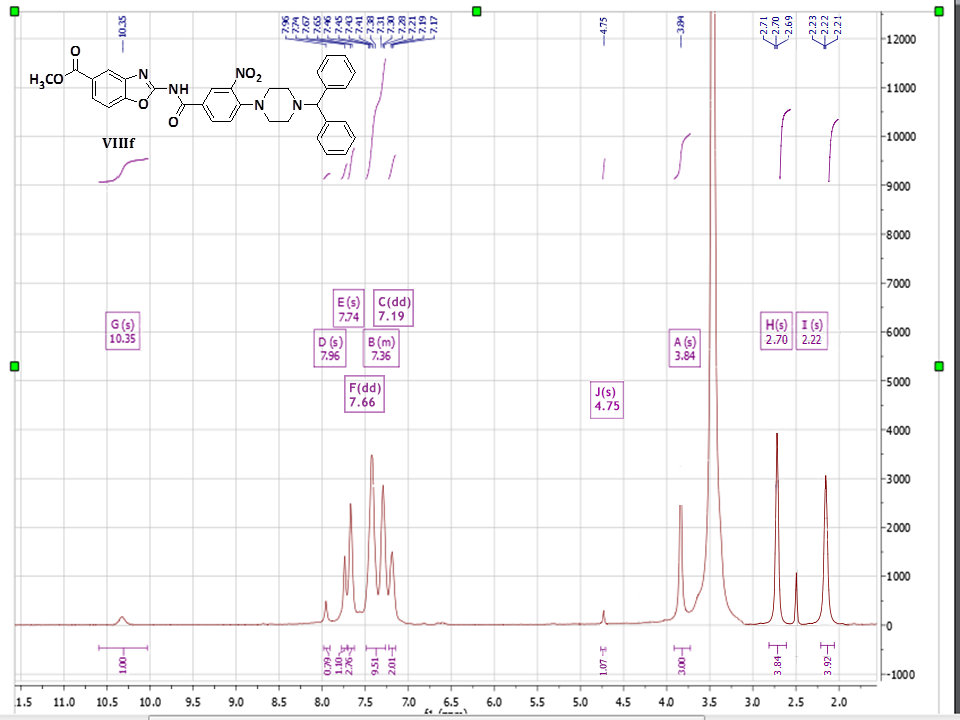
**

**Methyl 2-[N-(4-(4-methylpiperazin-1-yl)-3-nitrobenzamido)]benzo[*d*]oxazole-5-carboxylate (8g)**


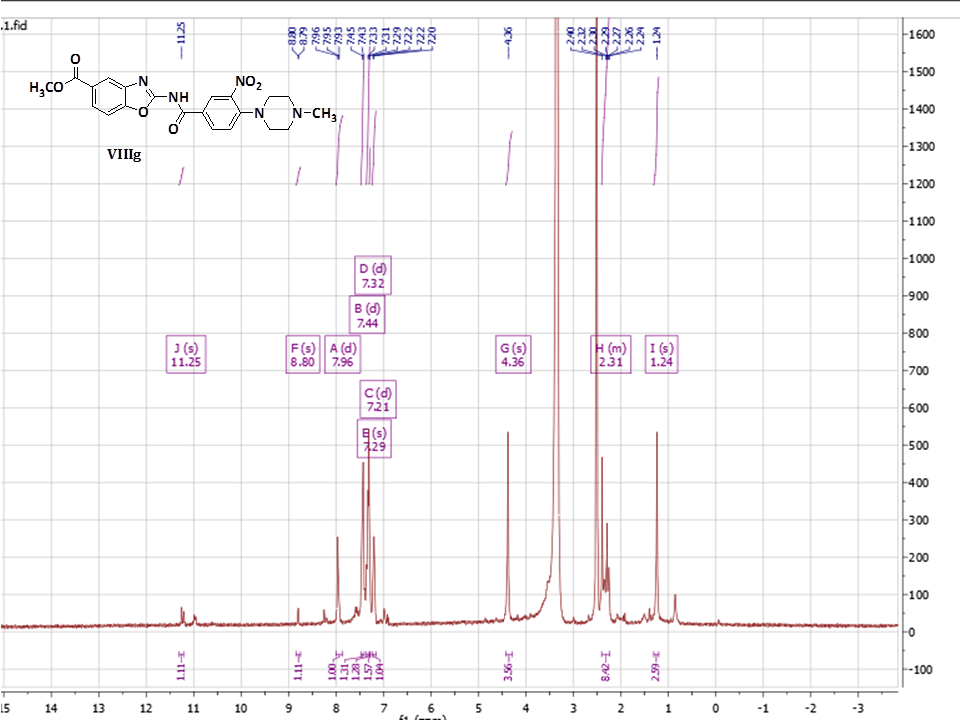

**Ethyl 2-[N-(3-nitro-4-(phenethylamino)benzamido)]thiazole-4-carboxylate (9a)**

**
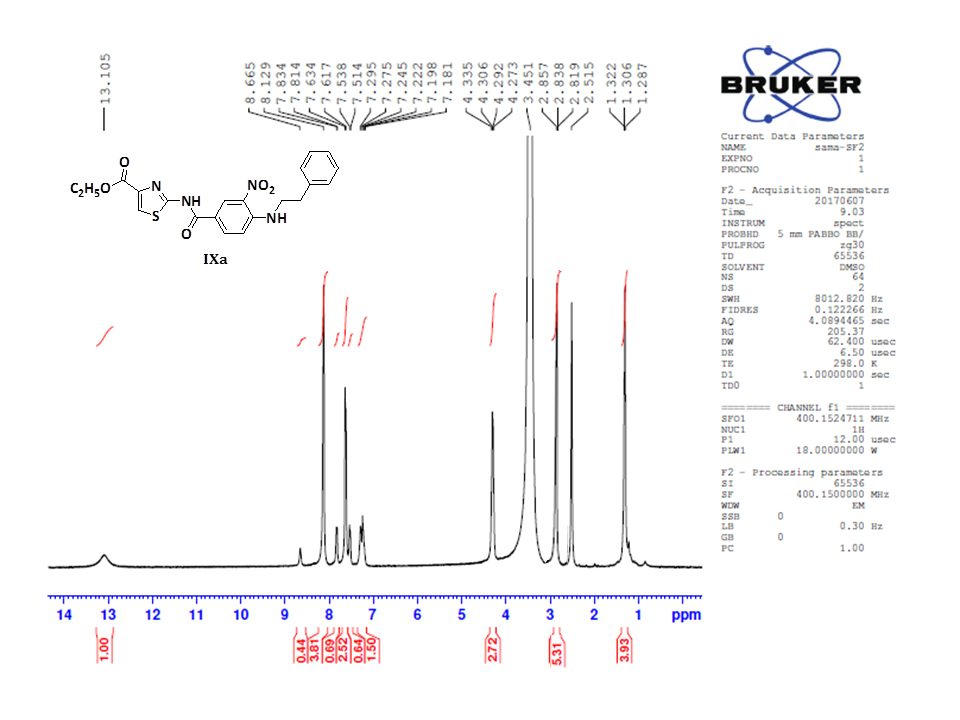
**

**Ethyl 2-[N-(4-benzylamino-3-nitrobenzamido)]thiazole-4-carboxylate (9b)**

**
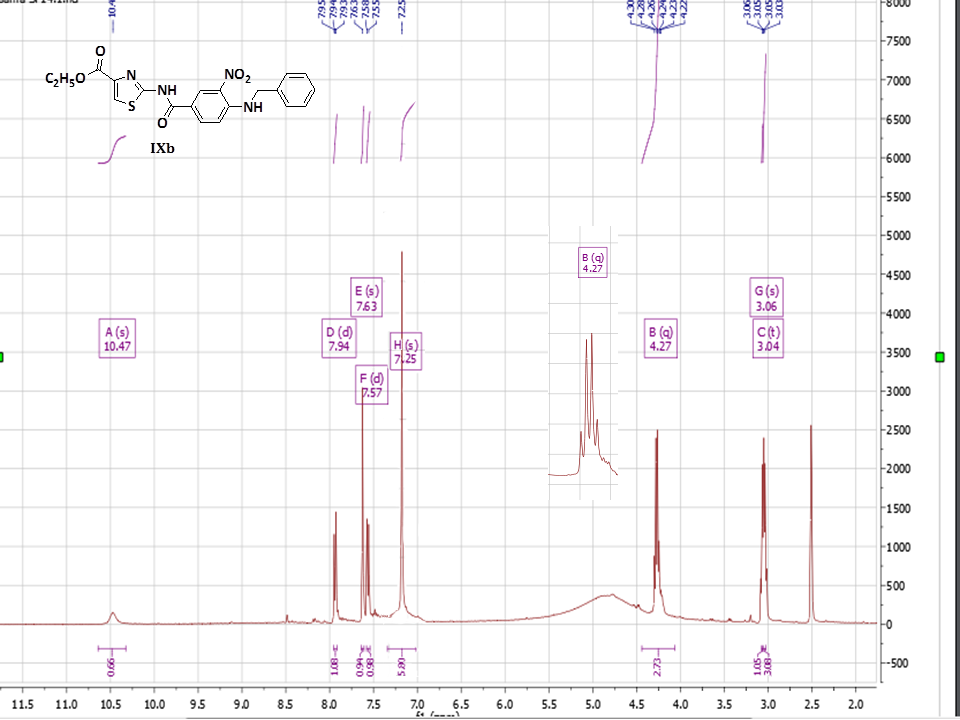
**

**Ethyl 2-[N-(4-(3,4-dichloroanilino)-3-nitrobenzamido)]thiazole-4-carboxylate (9c)**


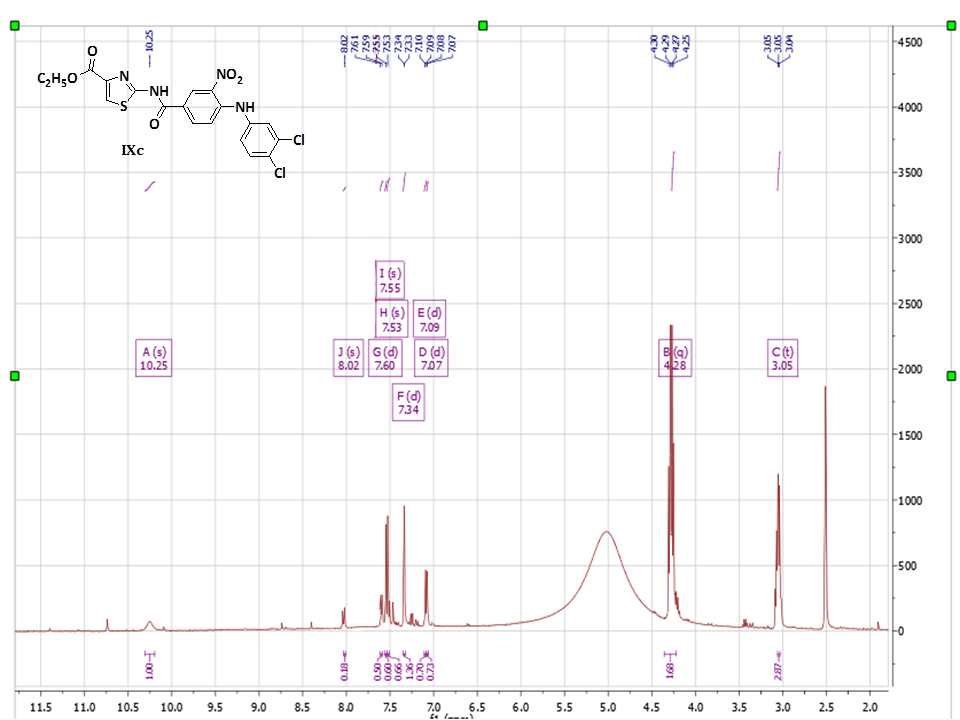

**Ethyl 2-[N-(4-(2-methyl-4-nitroanilino)-3-nitrobenzamido)]thiazole-4-carboxylate (9d)**

**
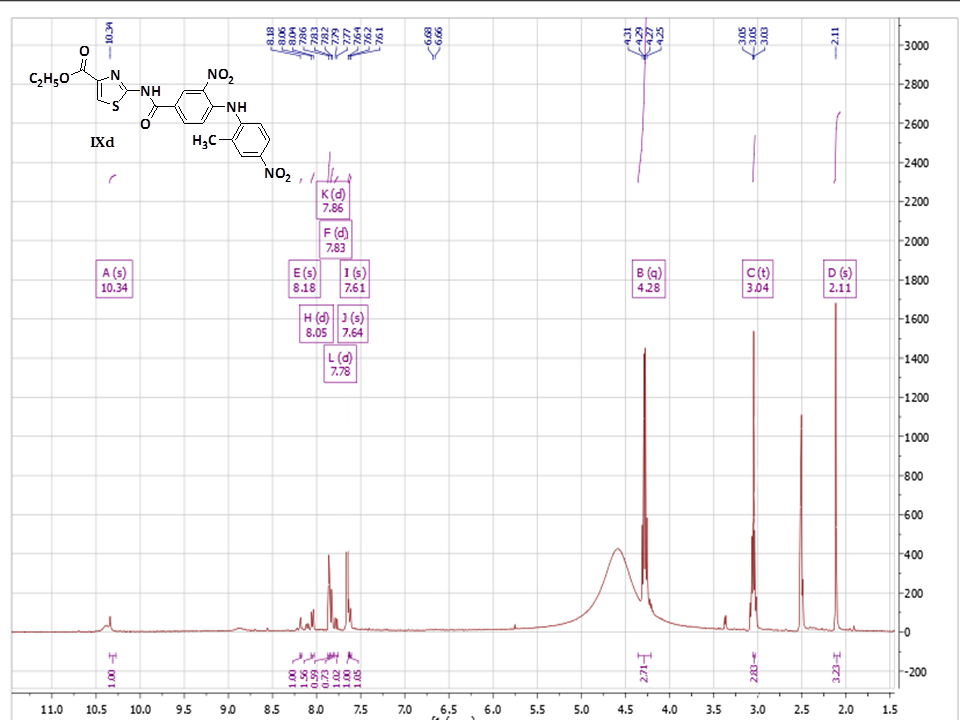
**

**Ethyl 2-[N-(4-cyclohexylamino-3-nitrobenzamido)]thiazole-4-carboxylate (9e)**


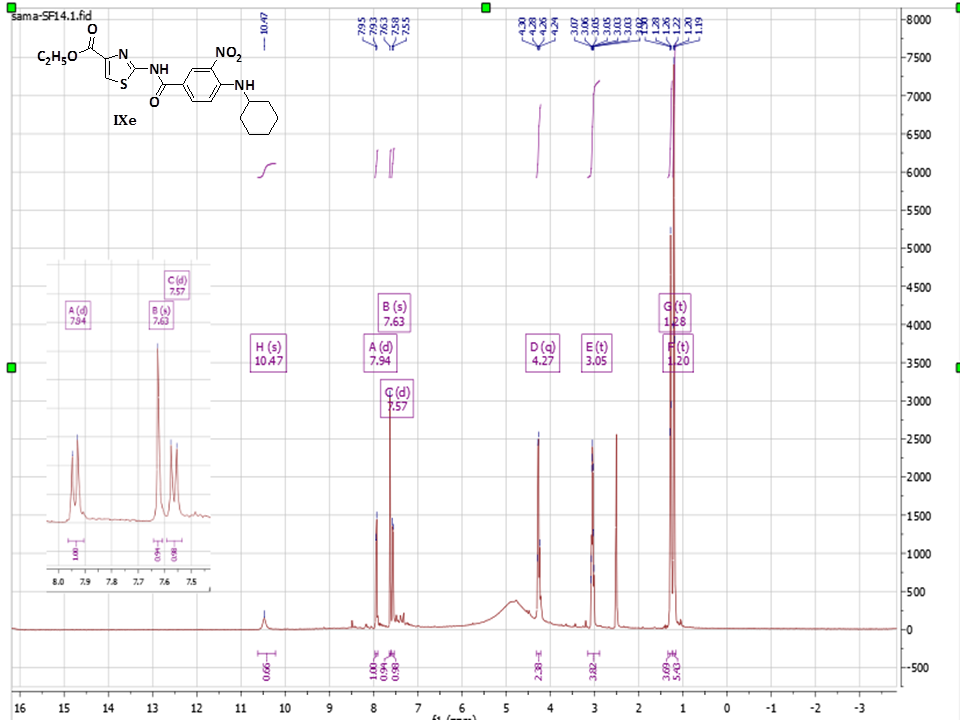

**Ethyl 2-[N-(4-(4-benzhydrylpiperazin-1-yl)-3-nitrobenzamido)]thiazole-4-carboxylate (9f)**


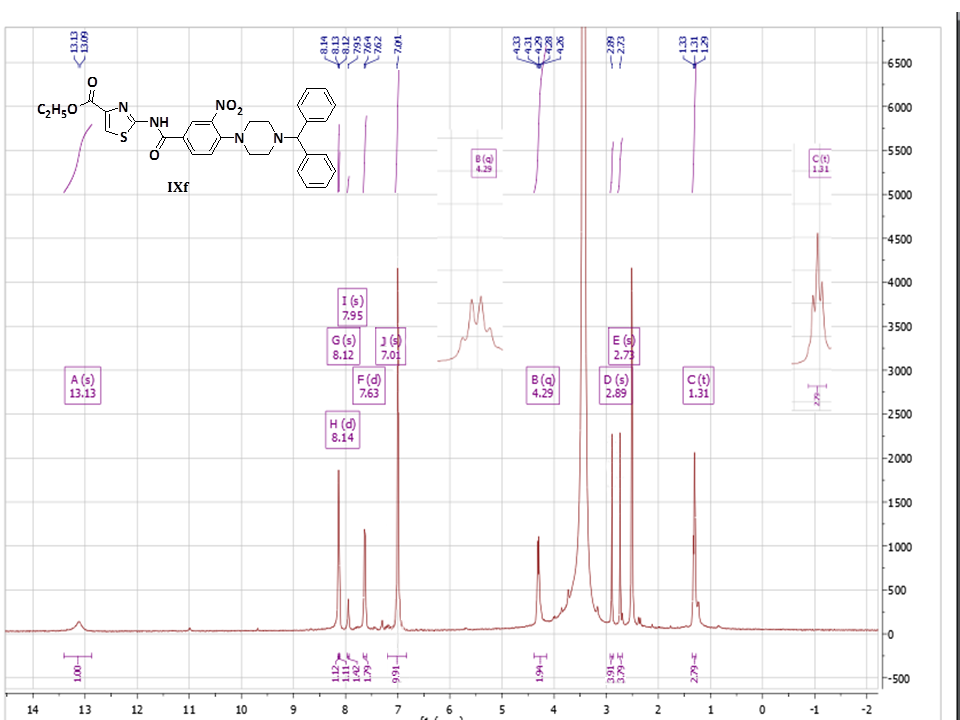

**Ethyl 2-[N-(4-(4-methylpiperazin-1-yl)-3-nitrobenzamido)]thiazole-4-carboxylate (9g)**


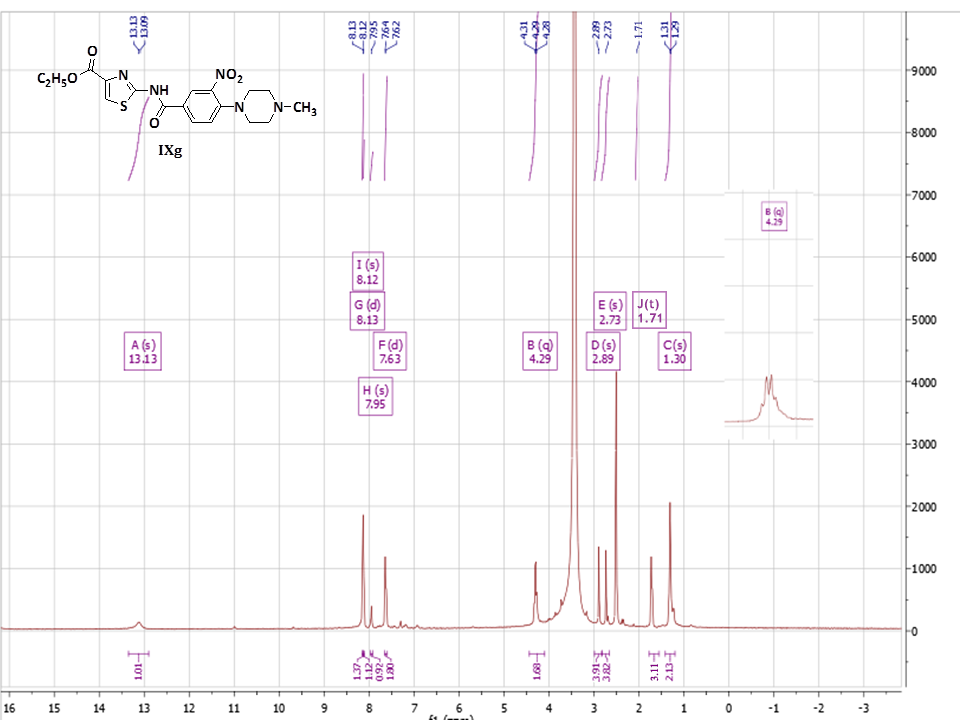

**Ethyl 2-[N-(3-nitro-4-(piperid-1-yl)benzamido)]thiazole-4-carboxylate (9h)**


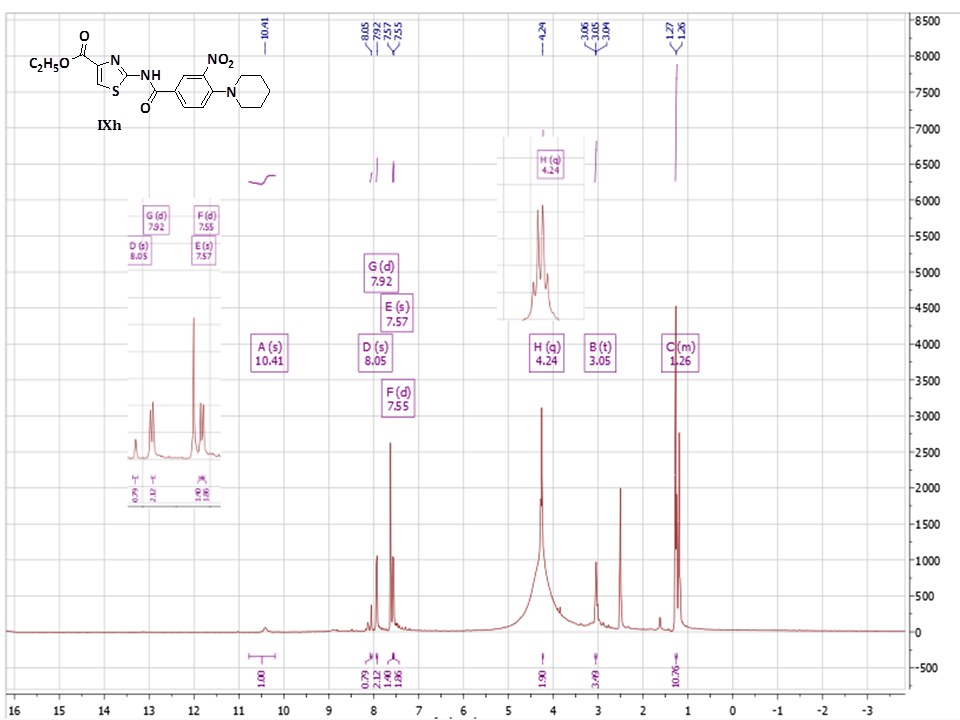

**2-[N-(4-(phenethylamino)-3-nitrobenzamido)]thiazole-4-carboxylic acid (11a)
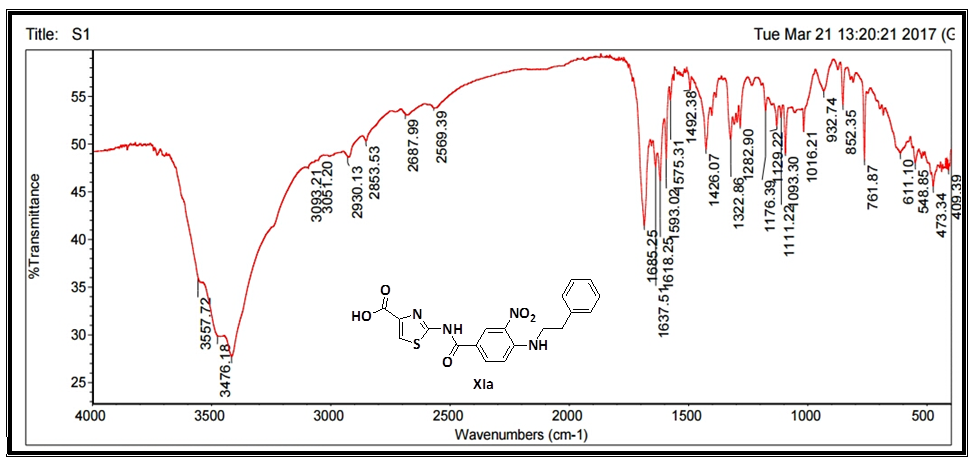
**

**5-(4-benzhydrylpiperazin-1-oyl)-2-[N-(4-(3,4-dichloroanilino)-3-nitrobenzamido)]benzo[*d*]oxazole (12c)**


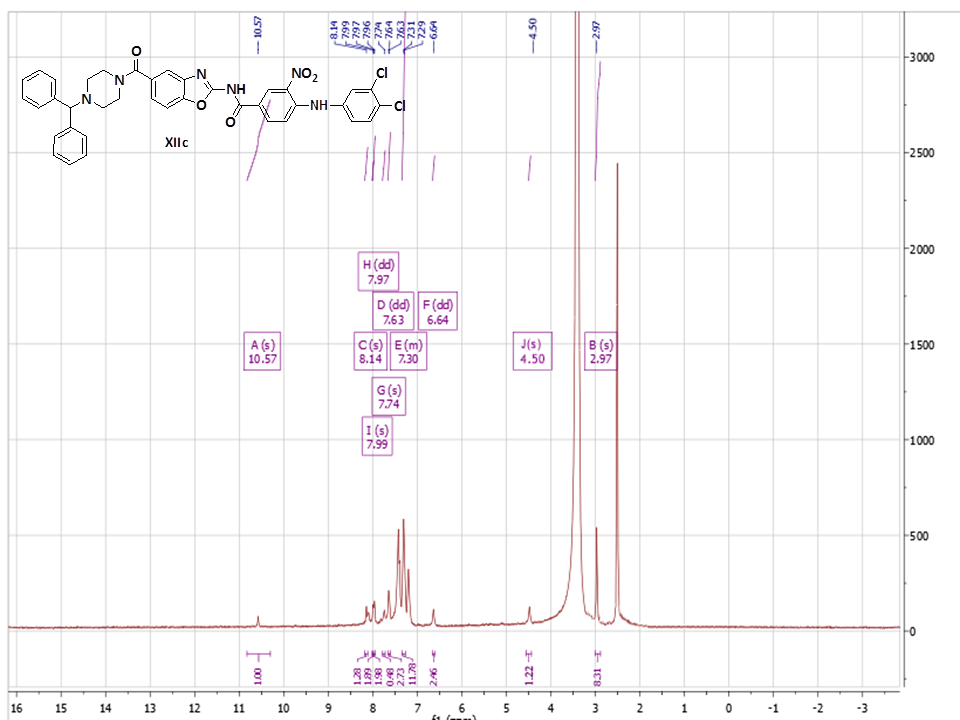

**5-(4-benzhydrylpiperazin-1-oyl)-2-[N-(4-(2-methyl-4-nitroanilino)-3-nitrobenzamido)]benzo[*d*]oxazole (12d)**

**
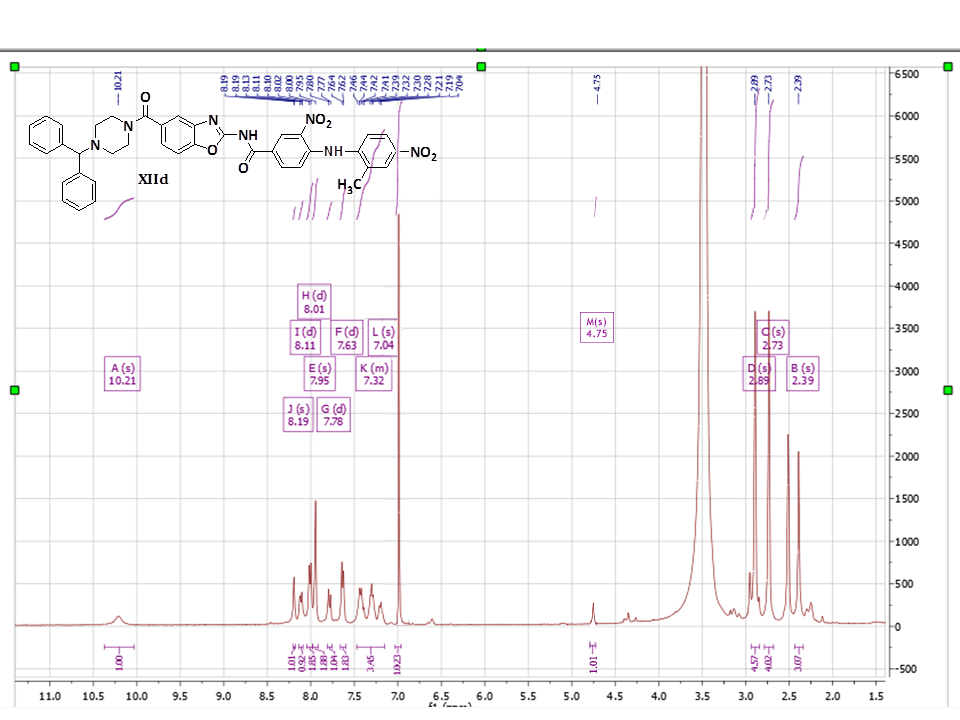
**

**5-(4-benzhydrylpiperazin-1-oyl)-2-[N-(4-cyclohexylamino-3-nitrobenzamido)]benzo[*d*]oxazole (12e)**

**
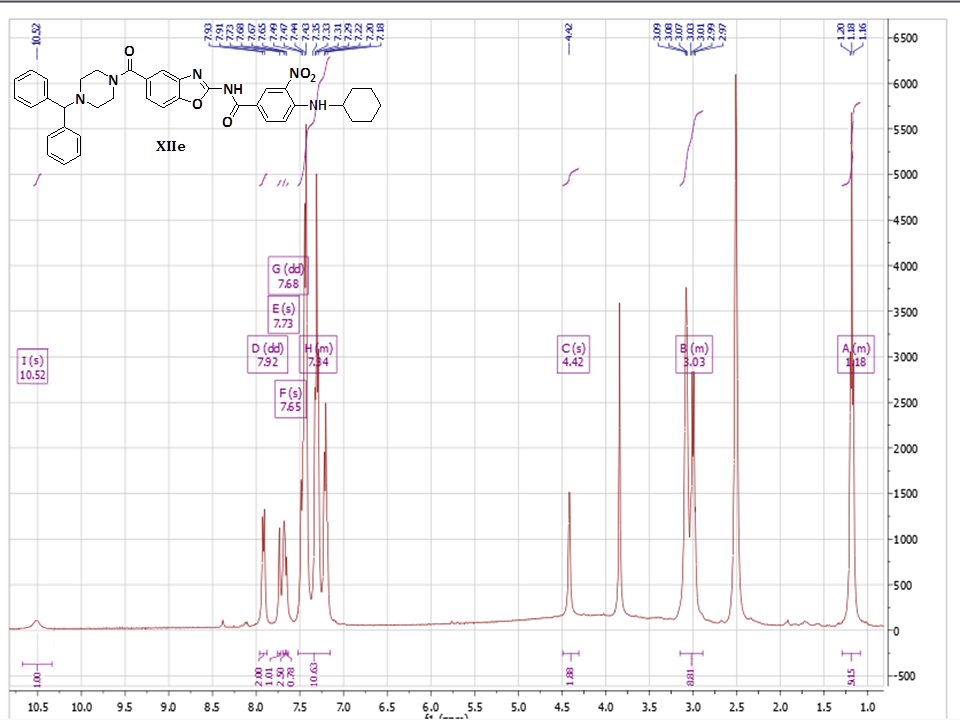
**

**4-(4-benzhydrylpiperazin-1-oyl)-2-[N-(4-(phenethylamino)-3-nitrobenzamido)]thiazole (13a)**

**
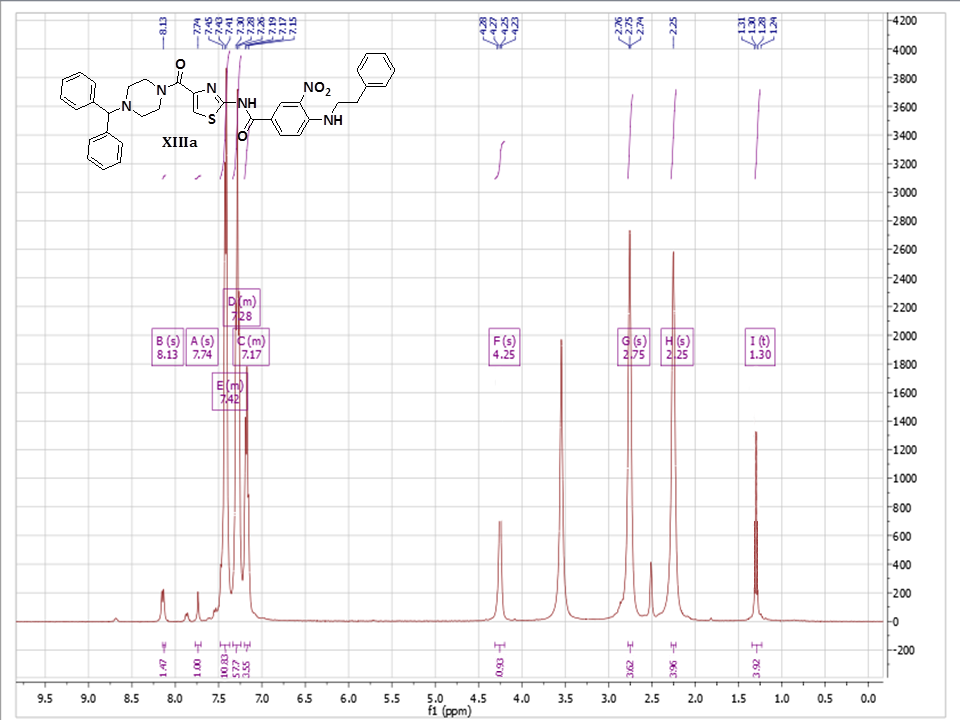
**

**4-(4-benzhydrylpiperazin-1-oyl)-2-[N-(4-(3,4-dichloroanilino)-3-nitrobenzamido)]thiazole (13c)**

**
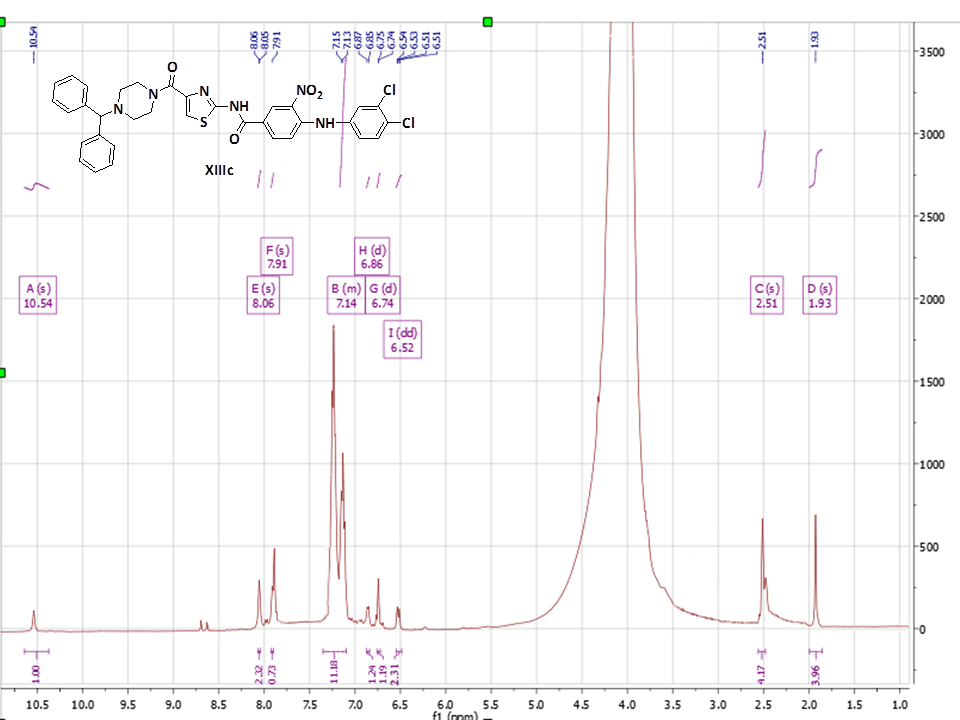
**

**4-(4-benzhydrylpiperazin-1-oyl)-2-[N-(4-(2-methyl-4-nitroanilino)-3-nitrobenzamido)]thiazole (13d)**

**
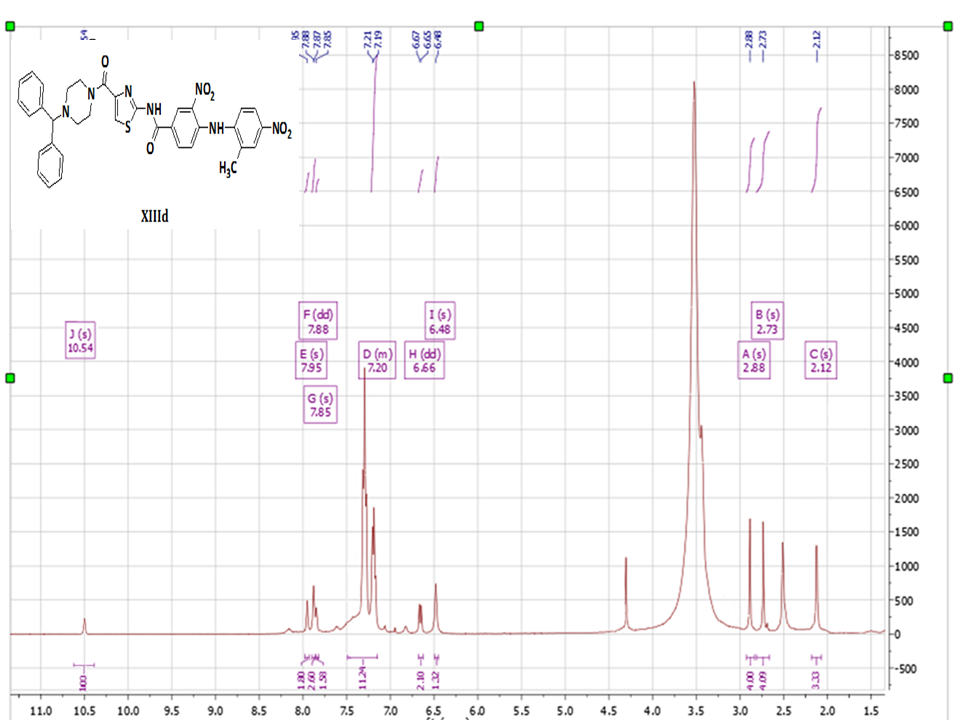
**

**4-(4-benzhydrylpiperazin-1-oyl)-2-[N-(4-cyclohexylamino-3-nitrobenzamido)]thiazole (13e)**

**
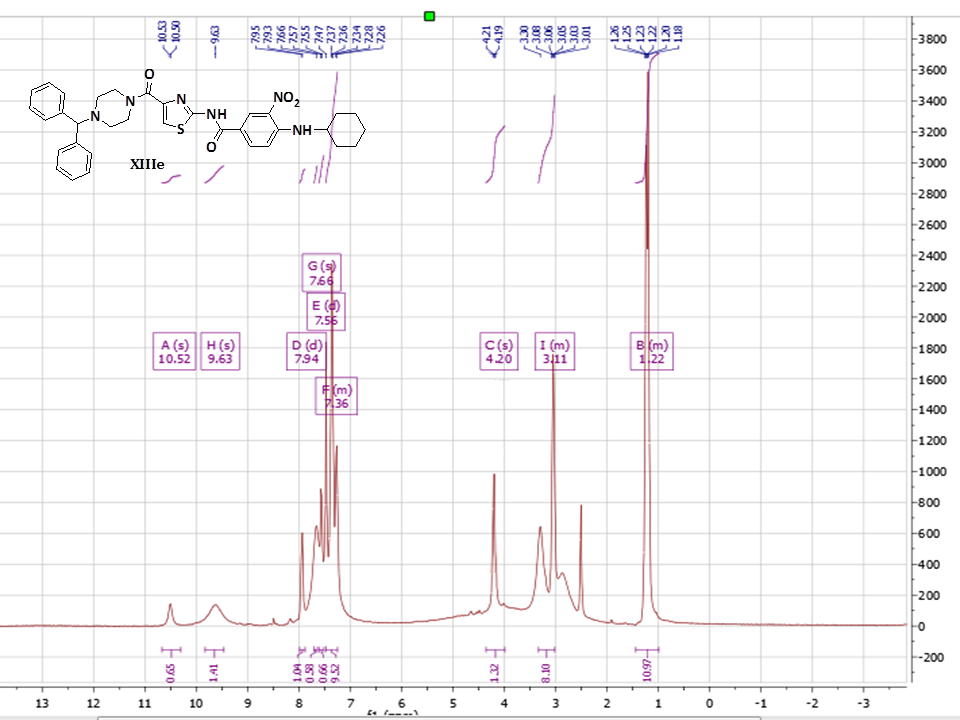
**

**Biological evaluation:**

### In vitro Anti-proliferative activity against 60 cell line panel

#### **Assay protocol:**

The human tumor cell lines of the cancer screening panel were grown in RPMI 1640 medium containing 5% fetal bovine serum and 2 mM L-glutamine. For a typical screening experiment, cells are inoculated into 96 well microtiter plates in 100 µL at plating densities ranging from 5,000 to 40,000 cells/well depending on the doubling time of individual cell lines. After cell inoculation, the microtiter plates are incubated at 37° C, 5 % CO_2_, 95 % air and 100 % relative humidity for 24 hrs prior to addition of experimental drugs. After 24 hrs, two plates of each cell line are fixed in situ with trichloroacetic acid (TCA), to represent a measurement of the cell population for each cell line at the time of drug addition (Tz). Experimental drugs are solubilized in dimethyl sulfoxide at 400-fold the desired final maximum test concentration and stored frozen prior to use. At the time of drug addition, an aliquot of frozen concentrate is thawed and diluted to twice the desired final maximum test concentration with complete medium containing 50 µg/mL gentamicin. Additional four, 10-fold or ½ log serial dilutions are made to provide a total of five drug concentrations plus control. Aliquots of 100 µl of these different drug dilutions are added to the appropriate microtiter wells already containing 100 µl of medium, resulting in the required final drug concentrations.

Following drug addition, the plates are incubated for an additional 48 hrs at 37°C, 5 % CO_2_, 95 % air, and 100 % relative humidity. For adherent cells, the assay is terminated by the addition of cold TCA. Cells are fixed in situ by the gentle addition of 50 µl of cold 50 % (w/v) TCA (final concentration, 10 % TCA) and incubated for 60 minutes at 4°C. The supernatant is discarded, and the plates are washed five times with tap water and air dried. Sulforhodamine B (SRB) solution (100 µl) at 0.4 % (w/v) in 1 % acetic acid is added to each well, and plates are incubated for 10 minutes at room temperature. After staining, unbound dye is removed by washing five times with 1 % acetic acid and the plates are air dried. Bound stain is subsequently solubilized with 10 mMtrizma base, and the absorbance is read on an automated plate reader at a wavelength of 515 nm. For suspension cells, the methodology is the same except that the assay is terminated by fixing settled cells at the bottom of the wells by gently adding 50 µl of 80 % TCA (final concentration, 16 % TCA).

#### **Data analysis**

Using the seven absorbance measurements [time zero, (Tz), control growth, (C), and test growth in the presence of drug at the five concentration levels (Ti)], the percentage growth is calculated at each of the drug concentrations levels. Percentage growth inhibition is calculated as:

- [(Ti-Tz)/(C-Tz)] x 100 for concentrations for which Ti>/ = Tz
- [(Ti - Tz)/Tz] x 100 for concentrations for which Ti <Tz^[[1]](#endnote-1)^

### Cell cycle analysis

This assay was carried out in The Research and Development Center, Faculty of Medicine, Al-Azhar University.

#### **Assay protocol:**

Cell cycle distribution was assessed using propidium iodide. Briefly, 1 × 105 cells were treated with PBS (as vehicle control) for 48 h. Next, cells were detached using 0.05 % trypsin for 5 min, harvested, and washed with PBS. Cells were then fixed with 75 % ethanol overnight. After centrifugation at 664×g for 15 min at 4 °C, the resulting supernatants were decanted. Cell pellets were stained with 10 µg/mL PI and 10 µg/mL RNase A in PBS buffer for 30 min at 37 °C in the dark. The samples were assayed using a FACScan flow cytometer and the results were analyzed using FlowJo v7.5.5 software.

**Annexin** **V-FITC assay**

This assay was carried out in The Research and Development Center, Faculty of Medicine, Al-Azhar University.

#### **Assay protocol:**

**A. Incubation of cells with Annexin V-FITC**

1-5 x 105 cells were collected by centrifugation. Cells were resuspended in 500 μl of 1X Binding Buffer. 5 μl of Annexin V-FITC and 5 μl of propidium iodide (PI 50mg/ml, optional.) were added and cells were incubated at room temperature for 5 min in the dark.

**B. Quantification by Flow Cytometry**

Annexin V-FITC binding was analyzed by flow cytometry (Ex = 488 nm; Em = 530 nm) using

FITC signal detector (usually FL1) and PI staining by the phycoerythrin emission signal detector (usually FL2).

For adherent cells, cells were gently trypsinized and washed once with serum-containing media before incubation with Annexin V-FITC.

### Detection of caspase-3 protein assay

This assay was carried out in The Research and Development Center, Faculty of Medicine, Al-Azhar University.

#### **Assay protocol**:

The Invitrogen Caspase-3 (active) Human kit is a solid phase sandwich Enzyme Linked Immuno-Sorbent Assay (ELISA). A monoclonal antibody specific fo human caspase-3 has been coated onto the wells of the microtiter strip provided. Samples, including a standard containing human active caspase-3, control specimens, and unknowns, are pipetted into these wells and then a rabbit antibody specific for human active caspase-3 is added to the wells. During the first incubation, the human caspase-3 protein binds to the immobilized (capture) antibody and the specific active caspase-3 antibody serves as a detection antibody by binding to the immobilized active caspase-3 protein. After the first incubation step and washing to remove excess protein and detection antibody, a horseradish peroxidase-labeled Anti-Rabbit IgG (Anti-Rabbit IgG HRP) is added. This binds to the detection antibody to complete the four-member sandwich. After a third incubation and washing to remove all the excess Anti-Rabbit IgG HRP, a substrate solution is added, which is acted upon by the bound enzyme to produce color. The intensity of this colored product is directly proportional to the concentration of human active caspase-3 present in the original specimen.

#### **Data analysis:**

The absorbance at 450 nm is measured having blanked the plate reader against a chromogen blank composed of 100 μl each of Stabilized Chromogen and Stop Solution. Then using curve fitting software, the standard curve is generated. A four-parameter algorithm provides the best standard curve fit. Read the concentrations for unknown samples and controls from the standard curve. Multiply value(s) obtained for sample(s) by the appropriate dilution factor to correct for the dilution. Samples producing signals greater than that of the highest standard should be diluted in Standard Diluent Buffer and reanalyzed.

### In vitro Bcl-2 activity

#### **Assay protocol:**

The assay was performed by TR-FRET technology using a recombinant BCL-2 and a peptide- ligand substrate. The TR-FRET signal from the assay is correlated with the amount of Ligand binding to BCL-2. Compounds were diluted in 100% DMSO then 10-fold dilution in 10% DMSO in 1X Reaction Buffer. 2 µl of the dilution was added to a 20 µl reaction so that the final concentration of DMSO is 1% in all of reactions. All of the binding reactions were conducted at room temperature. The 20 µl reaction mixture in Assay Buffer contains bcl-2, the indicated amount of the inhibitor, ligand, and the reaction dyes. The reaction mixture incubated for 180 min prior to reading the TR-FRET signal. For the background, ligand was replaced with assay buffer. Fluorescence signals for both the donor and acceptor dyes were measured using a Tecan Infinite M1000 plate reader. TR-FRET was recorded as the ratio of the fluorescence of the acceptor and the donor dyes (acceptor/donor).

#### **Data analysis:**

Binding experiments were performed in duplicate at each concentration. The TR-FRET data were analyzed using the computer software, Graphpad Prism. In the absence of the compound in wells containing BCL-2 ligand, the TR-FRET signal (Ft) in each data set was defined as 100% activity. In wells without peptide ligand, the TR-FRET signal (Fb) in each data set was defined as 0% activity. The percent activity in the presence of each compound was calculated according to the following equation: % activity = [(F- Fb)/(Ft - Fb)]×100, where F= the TR-FRET signal in the presence of the compound. The percent inhibition was calculated according to the following equation: % inhibition = 100 - % activity.

The values of % activity versus a series of compound concentrations were then plotted using non-linear regression analysis of Sigmoidal dose-response curve generated with the equation Y=B+(T-B)/1+10((LogIC_50_-X)×Hill Slope), where Y=percent activity, B=minimum percent activity, T=maximum percent activity, X= logarithm of compound and Hill Slope=slope factor or Hill coefficient. The IC50 value was determined by the concentration causing a half-maximal percent activity.

### PCR analysis and quantification of gene expression of Bax, Bcl-2, Bcl-xL

#### **Assay protocol:**

Primers should be designed according to standard PCR guidelines with a length of 18 to 25 nucleotides, and a GC content of 40% to 65%. Primer design should avoid internal secondary structure, and complementarity at the 3' ends within each primer and primer pair. Optimal results may require titration of primer concentration between 100 and 500 nM. A final concentration of 300 nM per primer is effective for most reactions. In general, reaction efficiency and/or specificity can be optimized using equal concentrations of each primer. For best results, amplicon size should be limited to 50–200 bp. Suggested input quantities of template are: 1 pg to 100 ng total RNA; 10 fg to 100 ng polyA(+) RNA.

First strand synthesis can be performed between 40°C and 52°C. Optimal results are generally obtained with a10-minute incubation at 50°C. Incubation at temperatures higher than 50°C can delay or eliminate the detection of some non-specific amplification artifacts.

However, this may also delay the Ct for detection of specific targets. Thaw all components, except the iScript reverse transcriptase, at room temperature. Mix gently, but thoroughly, and then centrifuge at 4°C to collect contents to the bottom of the tube. Chill on ice before using. Centrifuge again briefly at 4°C if needed.

Preparation of a reaction cocktail is crucial in quantitative PCR applications to reduce pipetting errors and maximize assay precision and accuracy. Assemble the reaction cocktail with all required components except sample template (total RNA) and dispense equal aliquots into each reaction tube. Add target sample to each reaction as the final step. Addition of sample as 5–10 μl volumes will improve assay precision. Replicate samples should be assembled as a master mix with a single addition of sample template.

### Determination of sample cytotoxicity on HCT-116 cells (MTT protocol)

**Assay protocol**

1-the 96 well tissue culture plate was inoculated with 1 X 105 cells / ml (100 ul / well) and incubated at 37°C for 24 hours to develop a complete monolayer sheet.

2- Growth medium was decanted from 96 well micro titer plates after confluent sheet of cells were formed, cell monolayer was washed twice with wash media.

3- two-fold dilutions of tested sample was made in RPMI medium with 2% serum (maintenance medium).

4- 0.1 ml of each dilution was tested in different wells leaving 3 wells as control, receiving only maintenance medium.

5- Plate was incubated at 37°C and examined. Cells were checked for any physical signs of toxicity, e.g. partial or complete loss of the monolayer, rounding, shrinkage, or cell granulation.

6- MTT solution was prepared (5mg/ml in PBS) (BIO BASIC CANADA INC).

8- 20ul MTT solution was added to each well. Place on a shaking table, 150rpm for 5 minutes, to thoroughly mix the MTT into the media.

9)  Incubate (37C, 5% CO2) for 4 hours to allow the MTT to be metabolized.

10)  Dump off the media. (dry plate on paper towels to remove residue if necessary.

11)    Resuspend formazan (MTT metabolic product) in 200ul DMSO. Place on a shaking table, 150rpm for 5 minutes, to thoroughly mix the formazan into the solvent.

12)   Read optical density at 560nm and subtract background at 620nm. Optical density should be directly correlated with cell quantity.


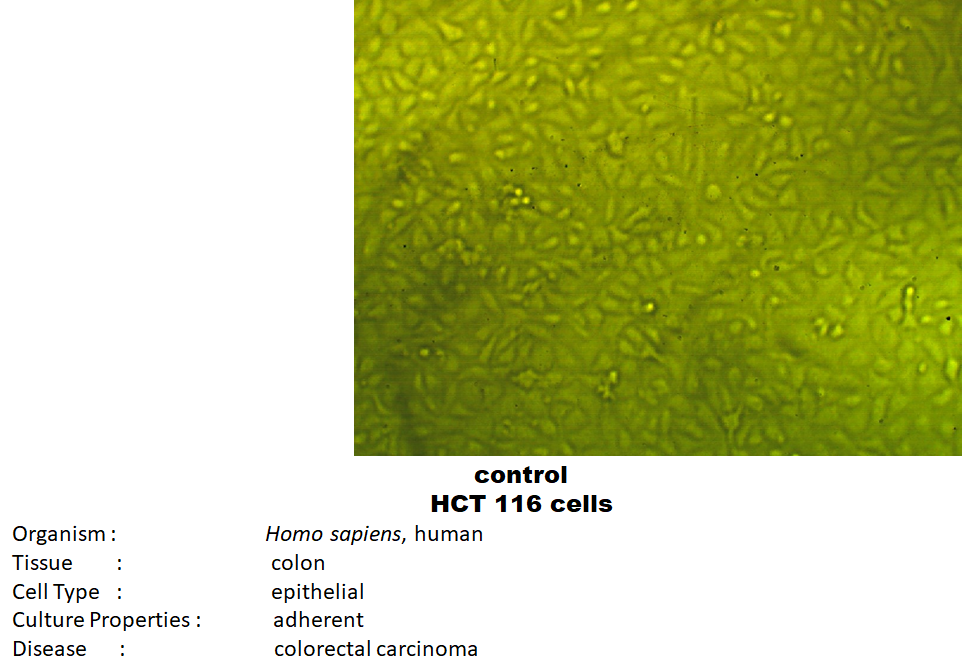


**Effect of representative target compounds on normal human cell lines**

***Assay protocol***

**1. Cell culture:**

Human fibroblast cell line was maintained in DMEM-high glucose supplemented with 100 µg/mL streptomycin, 100 units/mL penicillin and 10% heat-inactivated fetal bovine serum in a humidified, 5% (v/v) CO_2_ atmosphere at 37 ºC.

**2. Cytotoxicity assay**

The tested agent was dissolved in DMSO and kept at a stock concentration of 100*1000ug/ml.Cell seeding was done at a density of 2000 cells/ well in 96-well plates. Cells were exposed to different treatments for 72 h during which five different drug concentrations were tested. Cytotoxicity was assessed at the end of drug exposure using SRB assay as previously described [1]. Absorbance was measured at 545 nm using microplate reader (BioTek instruments, Vermont, USA). Results were expressed as the relative percentage of absorbance compared to control. Experiments were done in triplicates. Half-maximal inhibitory concentration (IC_50_), the drug concentration at which 50% growth inhibition is achieved, was calculated using GraphPad Prism software, version 5.00 (GraphPad Software, Inc. La Jolla, CA, USA).

**Results**

**In vitro Anti-proliferative activity against 60 cell line panel**


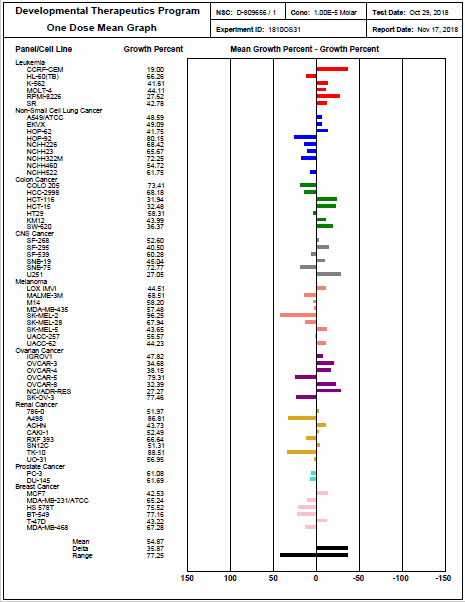


Figure S1. Mean graph of compound (8g) produced from NCI 60 cell line screening program; color codes are given for each cell line

| **Table S1: Cell growth inhibition percentage of NCI 60 cancer cell lines exhibited by investigated final compounds.**  **Cell line** | **Cell Growth Inhibition Percent for the tested compounds** | | | | | | | | | | | | | | | | | | | | | |
| --- | --- | --- | --- | --- | --- | --- | --- | --- | --- | --- | --- | --- | --- | --- | --- | --- | --- | --- | --- | --- | --- | --- |
|  | **8a** | **8b** | **8c** | **8d** | **8e** | **8f** | **8g** | **9a** | **9b** | **9c** | **9d** | **9e** | **9f** | **9g** | **9h** | **12c** | **12d** | **12e** | **13a** | **13c** | **13d** | **13e** |
| **Leukemia** | | | | | | | | | | | | | | | | | | | | | | |
| **CCRF-CEM** | 58.64 | 10.49 | 12.65 | 0 | 0 | 15.07 | 81.00 | 24.89 | 48.91 | 0 | 0 | 0 | 18.80 | 11.28 | 0 | 4.98 | 45.75 | 71.71 | 18.40 | 40.22 | 54.43 | 4.97 |
| **HL-60(TB)** | 13.55 | 4.91 | 2.77 | 0.31 | 0 | 0 | 33.74 | 40.63 | 3.53 | 0.01 | 0.47 | 1.31 | 6.60 | 0.27 | 0.77 | 0.35 | 8.24 | 17.95 | 5.97 | 14.11 | 5.33 | 1.50 |
| **K-562** | 8.16 | 0 | 53.04 | 0 | 0 | 29.54 | 58.39 | 55.74 | 0 | 3.33 | 0 | 0 | 42.44 | 55.03 | 0 | 2.88 | 45.08 | 52.99 | 44.62 | 46.79 | 37.55 | 16.05 |
| **MOLT-4** | --- | 0.86 | 3.53 | 0 | 0 | --- | 55.89 | --- | 0 | 0 | 0 | 0 | --- | 6.92 | 0 | 3.48 | 26.66 | 52.14 | --- | 32.11 | 36.23 | 22.12 |
| **RPMI-8226** | 31.62 | 0.20 | 11.36 | 0 | 0 | 25.56 | 72.38 | 43.70 | 20.06 | 0 | 0 | 0 | 40.03 | 21.49 | 0 | 10.57 | 60.82 | 59.96 | 43.55 | 46.66 | 37.67 | 35.81 |
| **SR** | --- | 0 | 9.67 | 0 | 0 | --- | 57.22 | --- | 0 | 0 | 0 | 0 | --- | 18.26 | 0 | 0 | 12.15 | 50.96 | --- | 32.33 | 26.68 | 9.78 |
| **Non-Small Cell Lung Cancer** | | | | | | | | | | | | | | | | | | | | | | |
| **A549/ATCC** | 11.60 | 3.73 | 9.59 | 0 | 0 | 11.13 | 51.41 | 2.85 | 1.86 | 0 | 0.95 | 0 | 14.90 | 0 | 2.18 | 9.25 | 37.81 | 43.88 | 26.45 | 27.60 | 36.80 | 6.85 |
| **EKVX** | 18.79 | 22.42 | 22.97 | 20.11 | 16.47 | 6.01 | 50.91 | 34.18 | 3.45 | 21.61 | 19.26 | 20.34 | 12.95 | 11.26 | 0 | 20.56 | 42.55 | 47.54 | 23.48 | 45.36 | 21.94 | 22.09 |
| **HOP-62** | 42.70 | 6.58 | 7.69 | 0 | 0 | 15.36 | 58.25 | 22.39 | 9.34 | 0 | 0 | 5.79 | 21.03 | 3.17 | 3.90 | 8.10 | 13.25 | 52.65 | 22.70 | 31.49 | 49.53 | 9.06 |
| **HOP-92** | 16.25 | 24.94 | 24.89 | 0 | 18.47 | 25.54 | 19.85 | 59.65 | 25.52 | 17.18 | 0 | 23.80 | 35.12 | 26.71 | 17.19 | 23.38 | 57.38 | 26.71 | 38.71 | 29.33 | 10.01 | 36.33 |
| **NCI-H226** | 15.18 | 25.13 | 19.26 | 7.91 | 28.76 | 17.32 | 31.58 | 42.86 | 20.62 | 27.96 | 12.72 | 46.30 | 27.91 | 26.63 | 16.72 | 22.88 | 20.14 | 34.86 | 29.93 | 27.33 | 20.91 | 40.00 |
| **NCI-H23** | 12.82 | 10.48 | 13.34 | 4.07 | 10.20 | 6.07 | 34.33 | 33.13 | 5.68 | 11.09 | 7.57 | 17.64 | 8.27 | 13.62 | 3.13 | 14.94 | 54.28 | 30.52 | 21.33 | 50.00 | 18.98 | 22.79 |
| **NCI-H322M** | 10.99 | 4.31 | 6.72 | 0 | 2.43 | 3.58 | 27.75 | 16.39 | 4.55 | 6.69 | 3.16 | 3.81 | 0 | 3.72 | 0.59 | 5.82 | 14.35 | 40.63 | 10.17 | 6.24 | 41.10 | 8.40 |
| **NCI-H460** | 34.19 | 0 | 3.43 | 0 | 0 | 4.63 | 45.28 | 7.90 | 0 | 2.12 | 0 | 0 | 9.35 | 0 | 1.12 | 7.78 | 20.16 | 53.23 | 24.19 | 19.19 | 61.85 | 0.44 |
| **NCI-H522** | 31.05 | 7.54 | 24.81 | 5.64 | 6.81 | 32.45 | 38.25 | 42.92 | 16.25 | 15.01 | 9.30 | 6.74 | 36.47 | 21.12 | 14.63 | 12.60 | 65.53 | 35.77 | 33.36 | 38.46 | 26.62 | 32.69 |
| **Colon Cancer** | | | | | | | | | | | | | | | | | | | | | | |
| **COLO 205** | --- | 0.24 | 3.79 | 0 | 0 | --- | 26.59 | --- | 12.60 | 0.36 | 0 | 4.03 | --- | 0.83 | 11.63 | 13.82 | 25.63 | 20.30 | --- | 3.77 | 47.54 | 0 |
| **HCC-2998** | 1.66 | 1.39 | 0 | 0 | 1.30 | 0 | 31.82 | 0 | 0 | 2.16 | 2.76 | 0 | 0 | 2.67 | 0 | 2.50 | 17.83 | 37.78 | 1.14 | 2.64 | 20.94 | 27.35 |
| **HCT-116** | 30.34 | 1.77 | 18.58 | 0 | 0 | 19.10 | 68.06 | 36.59 | 0 | 2.57 | 0 | 0 | 28.21 | 12.13 | 0.61 | 1.54 | 34.53 | 59.11 | 37.14 | 53.63 | 43.44 | 17.36 |
| **HCT-15** | 33.27 | 11.31 | 29.10 | 0 | 0 | 0 | 67.52 | 20.08 | 0 | 14.92 | 1.29 | 2.08 | 11.54 | 1.01 | 0 | 4.55 | 22.97 | 64.91 | 12.38 | 15.11 | 44.68 | 3.07 |
| **HT29** | 0 | 0.20 | 8.25 | 3.25 | 0 | 21.06 | 41.69 | 26.12 | 3.60 | 0 | 0 | 0 | 16.55 | 5.21 | 2.88 | 3.25 | 31.93 | 37.79 | 23.48 | 6.89 | 13.46 | 13.86 |
| **KM12** | 19.68 | 0 | 26.01 | 0 | 0 | 0 | 56.01 | 10.84 | 8.25 | 9.36 | 0 | 1.95 | 12.92 | 2.54 | 4.18 | 14.52 | 35.38 | 48.56 | 21.12 | 19.19 | 41.65 | 8.55 |
| **SW-620** | 15.94 | 0 | 13.41 | 0 | 4.89 | 1.51 | 63.63 | 13.60 | 6.46 | 5.74 | 0 | 4.11 | 0.84 | 9.06 | 8.63 | 17.30 | 25.20 | 55.68 | 12.86 | 14.02 | 21.97 | 8.65 |
| **CNS Cancer** | | | | | | | | | | | | | | | | | | | | | | |
| **SF-268** | 23.10 | 3.14 | 17.35 | 2.10 | 0 | 9.23 | 47.40 | 24.16 | 11.12 | 10.79 | 3.80 | 3.75 | 18.46 | 6.77 | 8.41 | 20.68 | 23.93 | 41.77 | 27.47 | 19.12 | 32.88 | 13.71 |
| **SF-295** | 45.44 | 13.44 | 7.81 | 1.18 | 4.04 | 3.82 | 60.50 | 59.68 | 0.12 | 3.92 | 3.39 | 4.88 | 21.12 | 18.56 | 0.13 | 0.77 | 16.61 | 57.91 | 22.82 | 52.08 | 58.23 | 30.08 |
| **SF-539** | 16.43 | 1.89 | 0 | 3.93 | 0 | 12.73 | 39.72 | 17.51 | 3.63 | 0.69 | 3.32 | 0 | 7.16 | 15.03 | 2.57 | 2.85 | 7.27 | 40.10 | 6.05 | 16.35 | 22.97 | 6.70 |
| **SNB-19** | 22.60 | 9.95 | 6.02 | 1.03 | 0 | 4.70 | 54.96 | 18.05 | 0 | 5.72 | 5.15 | 6.10 | 9.09 | 8.69 | 6.25 | 7.54 | 19.19 | 51.72 | 18.68 | 69.34 | 36.93 | 14.11 |
| **SNB-75** | 17.78 | 12.25 | 19.57 | 2.46 | 28.26 | 36.61 | 27.23 | 17.69 | 33.26 | 9.78 | 4.48 | 25.71 | 40.49 | 19.63 | 20.10 | 40.51 | 24.91 | 28.28 | 31.73 | 21.95 | 30.70 | 13.89 |
| **U251** | 43.74 | 5.33 | 7.15 | 0 | 2.05 | 15.35 | 72.95 | 29.18 | 2.26 | 4.42 | 0 | 1.44 | 16.20 | 11.47 | 0.07 | 12.64 | 46.32 | 67.46 | 16.42 | 19.89 | 55.31 | 16.45 |
| **Melanoma** | | | | | | | | | | | | | | | | | | | | | | |
| **LOX IMVI** | --- | 2.90 | 44.24 | 0.27 | 2.48 | --- | 55.49 | --- | 1.97 | 8.56 | 2.39 | 5.02 | --- | 5.10 | 0 | 9.28 | 20.03 | 54.54 | --- | 34.11 | 14.35 | 8.53 |
| **MALME-3M** | 12.14 | 14.60 | 0 | 0 | 6.88 | 35.50 | 31.49 | 6.33 | 2.34 | 0 | 0 | 3.51 | 12.01 | 4.44 | 1.63 | 6.58 | 42.84 | 31.27 | 5.59 | 14.88 | 0 | 10.61 |
| **M14** | 26.98 | 0 | 16.12 | 0 | 0 | 11.30 | 41.80 | 20.96 | 8.47 | 2.43 | 0 | 1.24 | 19.45 | 4.37 | 8.08 | 0 | 11.25 | 41.81 | 13.03 | 17.77 | 4.96 | 22.17 |
| **MDA-MB-435** | 14.90 | 1.55 | 12.18 | 0 | 1.14 | 0 | 42.52 | 17.35 | 9.98 | 2.57 | 1.25 | 5.37 | 0 | 5.09 | 5.72 | 7.69 | 23.47 | 35.09 | 11.67 | 16.08 | 8058 | 8.98 |
| **SK-MEL-2** | 28.12 | 0 | 7.26 | 0 | 0 | 12.12 | 3.75 | 22.52 | 5.22 | 0.04 | 0 | 2.16 | 0 | 0 | 0 | 0 | 3.86 | 9.15 | 20.63 | 0 | 4.32 | 0 |
| **SK-MEL-28** | 26.96 | 2.74 | 9.22 | 0 | 0 | 7.66 | 32.06 | 13.29 | 0 | 1.07 | 0 | 0 | 4.34 | 0 | 0 | 0.59 | 16.35 | 33.52 | 7.89 | 1.70 | 11.92 | 0 |
| **SK-MEL-5** | 23.79 | 7.10 | 13.64 | 6.45 | 3.08 | 6.08 | 56.35 | 35.67 | 1.11 | 10.30 | 0.34 | 20.83 | 6.52 | 4.19 | 0 | 3.85 | 38.32 | 50.35 | 12.88 | 52.72 | 14.51 | 40.42 |
| \| **UACC-257** \| \| --- \| | 20.12 | 0.72 | 1.34 | 0 | 1.86 | 8.35 | 44.43 | 35.78 | 5.66 | 0 | 0 | 2.40 | 7.99 | 0.57 | 6.21 | 6.39 | 17.60 | 37.81 | 9.01 | 12.76 | 12.93 | 10.57 |
| **UACC-62** | 31.32 | 8.72 | 48.63 | 0 | 10.55 | 16.25 | 55.77 | 42.23 | 6.38 | 18.14 | 4.14 | 16.15 | 29.27 | 18.02 | 6.53 | 20.73 | 44.95 | 54.18 | 33.10 | 35.02 | 12.90 | 32.85 |
| **Ovarian Cancer** | | | | | | | | | | | | | | | | | | | | | | |
| **IGROV1** | 25.72 | 5.01 | 21.30 | 0 | 23.95 | 9.94 | 52.18 | 24.53 | 9.51 | 18.49 | 1.79 | 33.48 | 19.15 | 19.28 | 4.65 | 21.18 | 26.05 | 48.91 | 30.57 | 17.60 | 34.11 | 20.41 |
| **OVCAR-3** | 27.46 | 0 | 3.23 | 0 | 0 | 3.72 | 65.32 | 16.33 | 5.64 | 0 | 0 | 3.88 | 10.41 | 0 | 1.20 | 16.97 | 50.27 | 47.16 | 7.06 | 6.85 | 21.92 | 7.81 |
| **OVCAR-4** | 28.65 | 4.43 | 9.89 | 0 | 0 | 16.28 | 61.85 | 38.03 | 13.39 | 5.09 | 0 | 0 | 27.28 | 24.06 | 0 | 26.54 | 51.18 | 61.16 | 38.59 | 56.20 | 37.15 | 31.13 |
| **OVCAR-5** | 0 | 6.03 | 17.84 | 0 | 0 | 2.20 | 20.69 | 8.92 | 0 | 5.05 | 0.35 | 0 | 0 | 2.66 | 3.13 | 10.04 | 14.45 | 27.62 | 0 | 5.13 | 10.16 | 10.82 |
| **OVCAR-8** | 21.92 | 0 | 2.88 | 0 | 4.87 | 13.77 | 67.61 | 29.51 | 0.44 | 0 | 0 | 0 | 10.69 | 7.09 | 2.22 | 8.70 | 28.93 | 62.20 | 23.99 | 28.92 | 34.97 | 19.60 |
| **NCI/ADR-RES** | 20.20 | 6.07 | 17.84 | 0 | 1.11 | 5.02 | 72.73 | 24.79 | 2.03 | 6.53 | 1.48 | 7.07 | 10.14 | 7.33 | 0 | 14.97 | 26.23 | 64.73 | 21.55 | 41.24 | 49.34 | 22.98 |
| **SK-OV-3** | 15.21 | 4.33 | 15.10 | 0 | 11.38 | 11.68 | 22.54 | 28.96 | 16.16 | 5.66 | 0 | 13.77 | 11.12 | 15.37 | 11.85 | 24.67 | 26.38 | 26.16 | 28.48 | 22.45 | 14.14 | 10.19 |
| **Renal Cancer** | | | | | | | | | | | | | | | | | | | | | | |
| **786-0** | 12.09 | 0 | 6.45 | 0.33 | 0 | 0.09 | 48.03 | 29.86 | 0 | 0 | 0 | 0 | 2.76 | 7.21 | 0 | 0 | 17.89 | 46.43 | 9.93 | 15.54 | 12.58 | 12.31 |
| **A498** | 25.47 | 0.33 | 5.26 | 0 | 0 | 7.75 | 13.82 | 52.32 | 1.06 | 8.42 | 11.58 | 0 | 4.13 | 12.04 | 0 | 21.33 | 40.32 | 23.78 | 9.90 | 11.48 | 7.51 | 12.40 |
| **ACHN** | 18.23 | 0 | 26.86 | 0 | 0.35 | 9.78 | 56.27 | 15.01 | 0 | 12.33 | 0 | 9.53 | 16.65 | 7.19 | 0 | 11.07 | 19.31 | 52.99 | 14.62 | 30.81 | 31.04 | 13.40 |
| **CAKI-1** | 16.34 | 12.31 | 38.95 | 10.47 | 15.54 | 16.72 | 47.51 | 22.69 | 17.55 | 16.32 | 7.40 | 18.14 | 26.50 | 12.04 | 16.06 | 40.66 | 30.57 | 45.47 | 21.13 | 29.22 | 36.30 | 21.65 |
| **RXF 393** | 5.24 | 29.07 | 0 | 0 | 3.26 | 0 | 33.36 | 14.28 | 0 | 7.87 | 2.33 | 5.20 | 0 | 7.19 | 0 | 6.42 | 24.35 | 25.12 | 12.57 | 16.22 | 5.85 | 14.98 |
| **SN12C** | 19.96 | 6.71 | 8.42 | 2.83 | 8.66 | 8.30 | 48.69 | 23.91 | 0 | 7.13 | 4.64 | 6.12 | 15.52 | 17.01 | 4.83 | 3.89 | 25.04 | 46.18 | 22.59 | 6.56 | 31.75 | 14.40 |
| **TK-10** | 0 | 0 | 0 | 0 | 0 | 0 | 11.49 | 7.67 | 0.64 | 0 | 0.73 | 0 | 0 | 1.81 | 0 | 0 | 9.07 | 15.49 | 0 | 28.45 | 0 | 0.01 |
| **UO-31** | 25.38 | 8.28 | 21.96 | 9.28 | 24.56 | 34.11 | 43.05 | 53.13 | 23.44 | 16.74 | 11.73 | 25.26 | 40.41 | 5.49 | 21.22 | 33.47 | 26.70 | 33.53 | 52.04 | 33.72 | 33.75 | 33.93 |
| **Prostate Cancer** | | | | | | | | | | | | | | | | | | | | | | |
| **PC-3** | 24.31 | --- | --- | --- | --- | 24.99 | 38.92 | 26.91 | 10.53 | --- | --- | --- | 44.26 | 12.81 | 7.44 | 17.31 | 34.38 | 46.05 | 41.54 | 22.65 | 36.49 | 23.93 |
| **DU-145** | 24.85 | 0 | 15.79 | 0 | 0 | 0.06 | 30.31 | 23.34 | 2.28 | 0 | 0 | 0 | 8.23 | 0 | 0 | 2.64 | 24.25 | 38.08 | 20.50 | 2.79 | 25.30 | 1.05 |
| **Breast Cancer** | | | | | | | | | | | | | | | | | | | | | | |
| **MCF7** | 24.73 | 13.38 | 16.24 | 2.63 | 13.72 | 13.82 | 57.47 | 38.13 | 1.65 | 12.28 | 6.90 | 5.64 | 28.08 | 15.92 | 0.22 | 30.52 | 30.90 | 49.78 | 23.88 | 33.26 | 32.33 | 18.74 |
| **MDA-MB-231/ATCC** | 2.65 | 9.87 | 9.41 | 0 | 13.80 | 1.13 | 34.76 | 35.63 | 0 | 2.79 | 0 | 19.26 | 3.16 | 16.23 | 0 | 19.30 | 21.13 | 24.98 | 38.25 | 19.93 | 19.76 | 29.48 |
| **HS 578T** | 11.39 | 10.53 | 21.74 | 6.81 | 9.33 | 15.82 | 24.48 | 0 | 5.64 | 14.63 | 7.83 | 15.18 | 13.53 | 5.74 | 9.45 | 19.83 | 24.12 | 24.03 | 13.20 | 9.17 | 17.44 | 9.19 |
| **BT-549** | 13.98 | 3.00 | 13.74 | 0 | 4.22 | 6.37 | 22.84 | 23.62 | 6.98 | 8.82 | 1.71 | 9.27 | 8.73 | 1.13 | 2.02 | 3.83 | 20.81 | 16.60 | 10.62 | 25.53 | 22.88 | 17.52 |
| **T-47D** | --- | 2.34 | 22.54 | 3.95 | 0 | --- | 56.78 | --- | 0 | 23.67 | 9.73 | 2.59 | --- | 19.72 | 0.03 | 31.28 | 77.92 | 55.74 | --- | 40.12 | 47.21 | 40.02 |
| **MDA-MB-468** | 0 | 25.96 | 4.54 | 0 | 4.05 | 0 | 32.72 | 16.45 | 1.88 | 8.89 | 0 | 17.16 | 9.10 | 0 | 0.51 | 15.11 | 74.53 | 24.84 | 0 | 78.30 | 10.28 | 0 |
| **Mean Growth %** | 79.89 | 94.54 | 85.97 | 101.16 | 97.81 | 89.53 | 54.87 | 73.78 | 94.52 | 94.05 | 100.33 | 94.28 | 85.35 | 90.59 | 97.82 | 88.59 | 70.02 | 57.71 | 80.14 | 74.59 | 70.75 | 84.16 |
| **Mean growth inhibition%** | 20.11 | 5.46 | 14.03 | -1.16 | 2.19 | 10.47 | 45.13 | 26.22 | 5.48 | 5.95 | -0.33 | 5.72 | 14.65 | 9.41 | 2.18 | 11.41 | 29.98 | 42.29 | 19.86 | 25.41 | 29.25 | 15.84 |

---: Not done

1. ### Table S2. Determination of sample cytotoxicity on HCT-116 cells (MTT protocol)


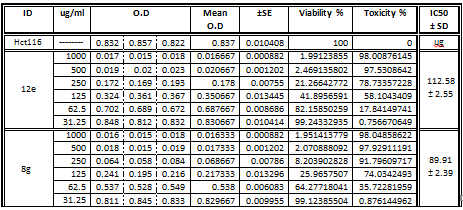


   **Figure S2**. Effect of **8g** and **12e** on HCT-116 cells at different concentrations


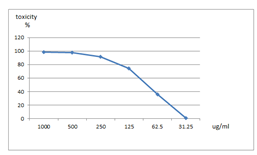


   **Figure S3**. Effect of **12e** on HCT-116 cells at different concentrations


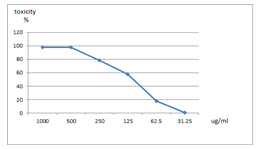


   **References**

   1. Tolba, M. F. & Abdel-Rahman, S. Z. (2015) Pterostilbine, an active component of blueberries, sensitizes colon cancer cells to 5-fluorouracil cytotoxicity, *Scientific reports.* **5**, 15239. [↑](#endnote-ref-1)
